# Generalization of DNA microarray dispersion properties: microarray equivalent of *t*-distribution

**DOI:** 10.1186/1745-6150-1-27

**Published:** 2006-09-07

**Authors:** Jaroslav P Novak, Seon-Young Kim, Jun Xu, Olga Modlich, David J Volsky, David Honys, Joan L Slonczewski, Douglas A Bell, Fred R Blattner, Eduardo Blumwald, Marjan Boerma, Manuel Cosio, Zoran Gatalica, Marian Hajduch, Juan Hidalgo, Roderick R McInnes, Merrill C Miller III, Milena Penkowa, Michael S Rolph, Jordan Sottosanto, Rene St-Arnaud, Michael J Szego, David Twell, Charles Wang

**Affiliations:** 1 McGill University and Genome Québec Innovation Centre, 740 Docteur Penfield Avenue, Montreal, Québec, H3A 1A4, Canada; 2 Human Genomics Laboratory, Genome Research Center, 52 Eoeun-dong, Yuseong-gu, Daejon, 305-333, Korea; 3 Transcriptional Genomics Core, Cedars-Sinai Medical Center, Los Angeles, CA 90048, USA; 4 Institut fur Onkologische Chemie, Heinrich Heine Universitat Dusseldorf, Moorenstr. 5, D-40225 Dusseldorf, Germany; 5 St. Luke's-Roosevelt Hospital Center and Columbia University, Molecular Virology Division, 432 West 58th Street, Antenucci Building, Room 709, New York, NY 10019, USA; 6 Institute of Experimental Botany AS CR, Rozvojová 135, CZ-165 02, Praha 6, Czech Republic and Charles University in Prague, Department of Plant Physiology, Viničná 5, 12844, Praha 2, Czech Republic; 7 Department of Biology, Higley Hall, 202 N. College Dr., Kenyon College, Gambier, OH 43022, USA; 8 Environmental Genomics Section, C3-03, PO Box 12233, National Institute of Environmental Health Sciences, Research Triangle Park, NC 27709, USA; 9 Department of Genetics, 425 Henry Mall, University of Wisconsin, Madison, WI 53706, USA; 10 Department of Plant Sciences, University of California, One Shields Ave, Davis, CA 95616, USA; 11 Department of Pharmaceutical Sciences, University of Arkansas for Medical Sciences, 4301 West Markham, Slot 522-3, Little Rock AR 72205, USA; 12 Respiratory Division, Department of Medicine, McGill University, Montreal, Quebec, Canada; 13 Department of Pathology, Creighton University School of Medicine, 601 North 30th Street, Omaha, NE, 68131-2197, USA; 14 Laboratory of Experimental Medicine, Department of Pediatrics, Faculty of Medicine and Dentistry, Palacky University in Olomouc, Puskinova 6, 775 20 Olomouc, Czech Republic; 15 Institute of Neurosciences and Department of Cellular Biology, Physiology and Immunology, Animal Physiology unit, Faculty of Sciences, Autonomous University of Barcelona, Bellaterra, Barcelona, 08193, Spain; 16 Programs in Genetics and Developmental Biology, The Research Institute, The Hospital for Sick Children, Toronto, Canada M5G 1X8; Departments of Molecular and Medical Genetics and Pediatrics, University of Toronto, Toronto, M5S 1A1, Canada; 17 Environmental Genomics Section, C3-03, PO Box 12233, National Institute of Environmental Health Sciences, Research Triangle Park, NC 27709, USA; 18 Section of Neuroprotection, Centre of Inflammation and Metabolism, The Faculty of Health Sciences, University of Copenhagen, Blegdamsvej 3, DK-2200, Copenhagen Denmark; 19 Arthritis and Inflammation Research Program, Garvan Institute of Medical Research, 384 Victoria St, Darlinghurst NSW 2010, Australia; 20 Department of Plant Sciences, University of California, One Shields Ave, Davis, CA 95616, USA; 21 Genetics Unit, Shriners Hospital for Children and Departments of Surgery and Human Genetics, McGill University, Montréal H3A 2T5, Québec, Canada; 22 Programs in Genetics and Developmental Biology, The Research Institute, The Hospital for Sick Children, Toronto, Canada M5G 1X8; Departments of Molecular and Medical Genetics, University of Toronto, Toronto, M5S 1A1, Canada; 23 Department of Biology, University of Leicester, LE1 7RH Leicester, UK; 24 Department of Medicine, Cedars-Sinai Medical Center, David Geffen School of Medicine, UCLA, Los Angeles, CA 90048, USA

## Abstract

**Background:**

DNA microarrays are a powerful technology that can provide a wealth of gene expression data for disease studies, drug development, and a wide scope of other investigations. Because of the large volume and inherent variability of DNA microarray data, many new statistical methods have been developed for evaluating the significance of the observed differences in gene expression. However, until now little attention has been given to the characterization of dispersion of DNA microarray data.

**Results:**

Here we examine the expression data obtained from 682 Affymetrix GeneChips^® ^with 22 different types and we demonstrate that the Gaussian (normal) frequency distribution is characteristic for the variability of gene expression values. However, typically 5 to 15% of the samples deviate from normality. Furthermore, it is shown that the frequency distributions of the difference of expression in subsets of ordered, consecutive pairs of genes (consecutive samples) in pair-wise comparisons of replicate experiments are also normal. We describe a consecutive sampling method, which is employed to calculate the characteristic function approximating standard deviation and show that the standard deviation derived from the consecutive samples is equivalent to the standard deviation obtained from individual genes. Finally, we determine the boundaries of probability intervals and demonstrate that the coefficients defining the intervals are independent of sample characteristics, variability of data, laboratory conditions and type of chips. These coefficients are very closely correlated with Student's *t-*distribution.

**Conclusion:**

In this study we ascertained that the non-systematic variations possess Gaussian distribution, determined the probability intervals and demonstrated that the *K*_*α *_coefficients defining these intervals are invariant; these coefficients offer a convenient universal measure of dispersion of data. The fact that the *K*_*α *_distributions are so close to *t-*distribution and independent of conditions and type of arrays suggests that the quantitative data provided by Affymetrix technology give "true" representation of physical processes, involved in measurement of RNA abundance.

**Reviewers:**

This article was reviewed by Yoav Gilad (nominated by Doron Lancet), Sach Mukherjee (nominated by Sandrine Dudoit) and Amir Niknejad and Shmuel Friedland (nominated by Neil Smalheiser).

## Open peer review

Reviewed by Yoav Gilad (nominated by Doron Lancet), Sach Mukherjee (nominated by Sandrine Dudoit) and Amir Niknejad and Shmuel Friedland (nominated by Neil Smalheiser). For the full reviews, please go to the Reviewers' comments section.

## Background

DNA microarrays provide large quantities of data for the study of diseases and biological processes in various organisms. However, microarray studies are subject to potential variations including biological and technical variability. Usually, the existence of a large dispersion makes it very difficult to draw any meaningful conclusions from the differences between the experimental and control groups [1,2]. Alison et al. [1] give the most recent general evaluation of the approaches and methods, summarizing the items where consensus has been established as well as outstanding questions; they underline the need for replicates and the usefulness of drawing information from neighboring genes ("shrinkage"), which is discussed at length here, provide the overview of clustering methods, etc. Many methods have been developed to deal with the problem of separation of systematic and random or pseudorandom components of the signal. For example, in the case of arrays using multi-probe sets, such as Affymetrix GeneChips^®^, we first have to derive a representative value of gene expression from the signals of individual probes ("low-level" analysis). The Affymetrix MAS 5 and GCOS use Tukey's biweight algorithm and yield an absolute expression value for each probe set (Affymetrix, 2005, GeneChip Expression Analysis Algorithm Tutorial, Part Number 700285, Rev. 1). The method of low-level analysis, developed by Li and Wong (dChip; [3,4]) is designed to assess the observed differences in expressions of genes on the arrays under comparison. It is based on fitting data to a simplified model, assuming that the noise variable is independent of the signal. A different model, called Robust Multiarray Analysis (RMA), was proposed by Speed, Bolstad, Irizarry and co-workers [5-7] (see also Bolstad, B.M., 2004, PhD Thesis, University of California, Berkeley). It uses a log-transform of the data implicitly assuming that the error is proportional to the signal intensity. In reality, the error variable has both, constant and proportional components. Once the representative value of the gene expression is known, standard statistical methods of comparison can be used for "high level" analysis of the observed differences. Nonparametric methods, such as the Mann-Whitney U-test (Wilcoxon test) or analysis of variance on ranks, are generally preferable, although the parametric *t*-test and ANOVA are also frequently used. It should be pointed out that the statistical methods can only separate the systematic variations from the random or pseudo-random component. Random errors are recognizable because they conform to some known frequency distribution, usually Gaussian distribution. However, occasionally, one or several samples exhibit spurious differences from the rest of the data, due to changes in the biological state of the examined cells, quality of RNA etc. Such undesirable effects are often significant and can be detected only by detailed comparisons of the individual replicate samples.

So far, very little attention has been given to the general properties of the dispersion of gene expression levels. With respect to applicability of various statistical methods it is useful to know how the standard deviation behaves across the expression range and whether this behavior is consistent from one assay to another and among the different types of arrays. Verification of normality of the frequency distribution of random fluctuations is particularly relevant. All parametric methods are based on concordance of the observed frequency distribution with the normal (Gaussian) distribution. Most physical and chemical systems, where random variations result mainly from collective interactions of large ensembles of particles, exhibit frequency distributions close to the Gaussian. The underlying mechanisms of microarray data variability are certainly of the same nature as the collective phenomena in physical systems but the ensemble of the processes involved is so complex that one would expect some compound distribution, far from the simple form expressed by the Gaussian prototype.

The object of the present study is to examine the frequency distributions, general properties of the standard deviations and coefficients of the probability intervals. It was found that the general characteristics of dispersion are useful for quality control, reduction of a system dimension and other purposes. Firstly an overview of the frequency distributions is given for both replicate arrays (five or more replicates) and consecutive sampling of the expression difference in the ordered pairs of genes in two-array comparisons. Subsequently, we describe the consecutive sampling analysis and evaluation of the linear characteristic function, approximating the standard deviation of the data variability across the arrays. The standard deviation function is then employed to define the probability intervals encompassing specific percentages of the observed values. The boundaries of these intervals are defined by probability coefficients *K*_*α*_. It was found that the values of *K*_*α *_coefficients obtained using various arrays are, at least in the first approximation, invariant. Finally, we compare the probability of coefficients *K*_*α *_with the corresponding values of inverse *t-*distribution.

## Results

In the present investigation we analyzed 682 Affymetrix microarrays of 22 different types. Our main objective was to study the microarray data derived from particular biological investigations, generated in many different microarray core laboratories, rather than the sets of arrays produced in the context of technology development or testing methods of analysis. Only a few "testing" sets were included. We evaluated the CEL files using MAS 4 (Affymetrix, 2002, Statistical Algorithm Description Document. Part Number 701137, Rev. 3.) and employed the "Average Difference" as expression signal value. Because MAS 5 and GCOS distort the frequency distributions in the near-zero region by ignoring the negative values, MAS 5 and GCOS outputs are not suitable. Prior to the analysis, we verified the linearity and quality of the data, in particular, the absence of clusters with significantly different expressions. All data on each array were normalized to 100% of the array mean; all Affymetrix control genes were excluded.

### Frequency distributions

In the case of experiments with five or more replicates, we tested the distributions of the expressions of individual genes. In addition, in all pair-wise comparisons we performed the Kolmogorov-Smirnov normality test on consecutive samples (Table 1). Based on our several thousands of tests, it was found that the Gaussian distribution was characteristic of the expression data obtained using the Affymetrix GeneChips^®^. Typically, for good-quality data, between 85 and 95 percent of samples passed the test. Moreover, a limited number of tests using the data obtained from fiberoptic bead-based oligonucleotide microarrays by Illumina led to the same conclusion [8].

**Table 1:** Illustration of the consecutive sampling procedure

Rank	Probe set	Sample Y1	Sample Y2	Y2-Y1	(Y2+Y1)/2	Sample Mean	SD (Y2-Y1)	SD(Y1)+ SD(Y2)
	...	...	...	...	...			
251	J03040_at	628	614	-14	621	614.4	71.1	71.8
252	M26880_at	657	583	-74	620			
253	HG384-HT384_at	577	662	86	619			
254	X04654_s_at	633	604	-29	619			
255	J04046_s_at	554	680	126	617			
256	X69908_rna1_at	593	640	47	617			
257	D85758_at	672	555	-117	614			
258	L12168_at	633	592	-41	612			
259	HG1614-HT1614_at	590	633	43	611			
260	X71428_at	571	649	77	610			
261	S75463_at	602	615	13	608			
262	X69910_at	579	630	50	604			

263	X57346_at	597	610	13	603	590.1	136.2	137.0
264	U01691_s_at	576	630	54	603			
265	X17620_at	605	594	-11	600			
266	U10323_at	562	617	56	590			
267	AJ001421_at	413	766	354	589			
268	X62654_rna1_at	576	602	26	589			
269	D64142_at	666	510	-156	588			
270	D21063_at	562	613	51	588			
271	X16560_at	588	580	-8	584			
272	D26600_at	580	586	6	583			
273	M19267_s_at	599	566	-33	583			
274	J02621_s_at	688	475	-213	582			

...	...	...	...	...	...			

Rank shows the rank from the highest mean expression. The columns "Sample Y1 and Y2" give the expression values, *Y*_2_* - Y*_1 _is the expressions difference and (Y2+Y1)/2 the mean expression of the probe sets Y1 and Y2. Sample Mean is the mean expression of the sample, "D(Y2-Y1)" is the standard deviation obtained from the difference of expressions and SD(Y1)+SD(Y2) is the sum of the standard deviations calculated from the values Y1 and Y2, respectively. The first 250 probe pairs are excluded to keep variation of the mean expression within the sample small.

**Figure 1:**
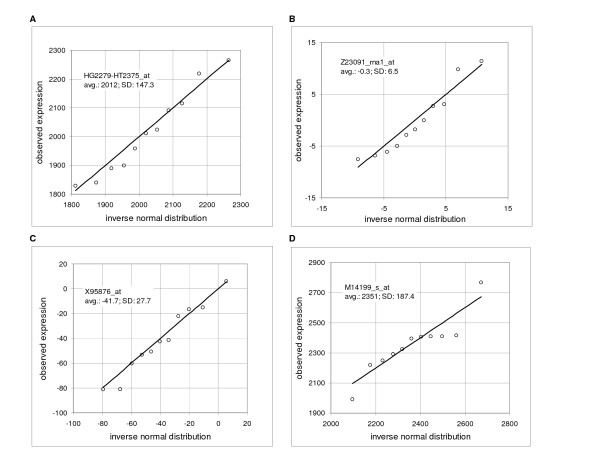
**Comparison of the observed frequency distribution to the inverse normal cumulative distribution**. Quantile-quantile plots show on y-axis the observed expression and on x-axis value of the corresponding inverse normal cumulative distribution. Microarray data are derived from HuGeneFL, using IMR90 cell line with 11 samples. Panels show the probe sets with the Kolmogorov-Smirnov maximum distance D equal or close to the mean value in the specified average expression rage. Inserts provide the Affymetrix probe set identification, average expression for a given gene and standard deviation. **A**: probe set HG2279-HT2375_at, rank 43, expression range from 1000 to 6681 (high range, maximum), average D in the range is 0.176, sample D is 0.176; **B**: probe set Z23091_rna1_at, rank 5484, expression range from -0.4 to 0.4 (near-zero range), average D in the rang is 0.181, sample D is 0.182; **C**: probe set X95876_at, rank 7003, expression range from -20 to -923 (negative range, minimum), average D in the range is 0.183, sample D is 0.182; **D**: example of the probe set that failed the test – probe set M14199_s_at, rank 25, sample D is 0.204 (data Novak et al., IMR90 [10]).

For illustration, Table 2 shows the results of the Kolmogorov-Smirnov test for six studies using Affymetrix GeneChips^® ^with five to 11 replicates and two studies using Illumina arrays with four replicates each. The mean percentage of probe sets across the arrays failing the Kolmogorov-Smirnov test was 6.9 using the algorithm of Sokal and Rohlf [9] (intrinsic hypothesis, P = 0.05). Usually, but not always, it was found that larger percentages of failures occur in the near-zero region. We did not examine systematically reasons for the failures, but it was often noted that there were outliers and, occasionally, a change of the slope or a discontinuity, noticeable in the quantile-quantile plots. Generally, we performed our analysis in the positive range of values above a small, arbitrary threshold. However, in the several tests, the percentage of failures above and below the threshold was practically identical. Figure 1 illustrates the similarity of the normal distribution and distribution of the expressions measured by typical probe sets in the human cell line IMR90 (11 replicates) in the high range (from 1000 to maximum of 6681, panel A), near-zero range (from -0.4 to 0.4, panel B) and negative range (from a minimum of -923 to -20, panel C; data Ref. [10]). A "typical" probe set is defined as a probe set with the Kolmogorov-Smirnov distance *D *at or close to the mean *D *in a given range. The figures show quantile-quantile plots (Q-Q plots), comparing the observed expression values to the corresponding values of the inverse normal cumulative distribution. The last panel D shows one sample that failed the test.

**Table 2:** Percentage of samples failing the Kolmogorov-Smirnov normality test

Array	Materials	No. of arrays	No. of probe sets	Threshold	% failure (total)	% failure (above)	% failure (below)
Affy. HuGeneFL	human cell line SKBR [a]	5	7070	2.7	7.2	6.9	7.8
Affy. HuGeneFL	human cell line IMR90 [a]	11	7070	4.1	6.3	6.5	5.9
Affy. U74Av2	murine lung tissue [b]	5	12422	10.0	6.1	6.6	5.5
Affy. U74Av2	murine lung tissue [c]	5	12422	13.6	7.6	8.3	6.4
Affy. U74Av2	murine lung tissue [d]	11	12422	14.2	10.6	10.6	10.7
Affy. Focus	human blood cell line [e]	9	8746	5.0	6.14	6.14	6.14
Illumina 1	human cell line GM10469 [f]	4	633	2.1	4.6	3.9	6.2
Illumina 2	human cell line GM10469 [f]	4	633	3.6	6.5	6.6	6.2
Average	---	---	---	---	6.9	6.9	6.9

Percentage of samples failing the Kolmogorov-Smirnov normality test at the level P = 0.05. All arrays are normalized to 100% of the mean value. The columns %failure (above) and %failure (below) give percentage of failures above and below the specified threshold.

[a] data Ref. [10].

[b] C57BL/6 (B6) WT mice, data Ref. [15].

[c] C57BL/6-*Cftr*-/- KO inbred mice, data Ref. [15].

[d] data M. Cosio.

[e] data O. Modlich and S. Raschke.

[f] lymphoblast cell line GM10469 [8].

**Table 3:** Comparison of the coefficients of standard deviation function derived from the consecutive sampling and individual probe sets

Array	No. of samples	Pair-wise a1	Individual genes a1	Difference %	Pair-wise a2	Individual genes a2	Difference %
HuGene FL (IMR90)	11	6.0	5.9	1.8	0.082	0.076	7.3
Focus	9	2.9	2.9	1.7	0.153	0.154	-0.6
MG-U74Av2	11	5.1	4.4	12.8	0.161	0.136	15.6
Illumina 1	4	2.7	2.4	12.2	0.092	0.085	7.7
Illumina 2	4	2.2	2.1	2.6	0.096	0.082	14.7
mean difference %	---	---	---	6.2	---	---	9.0

Columns pair-wise a1 and pair-wise a2 are the coefficients of the standard deviation characteristic function derived from the consecutive sampling. Columns individual genes a1 and a2 show the values derived from the individual probe sets and difference is the difference in % between the two methods.

**Figure 2:**
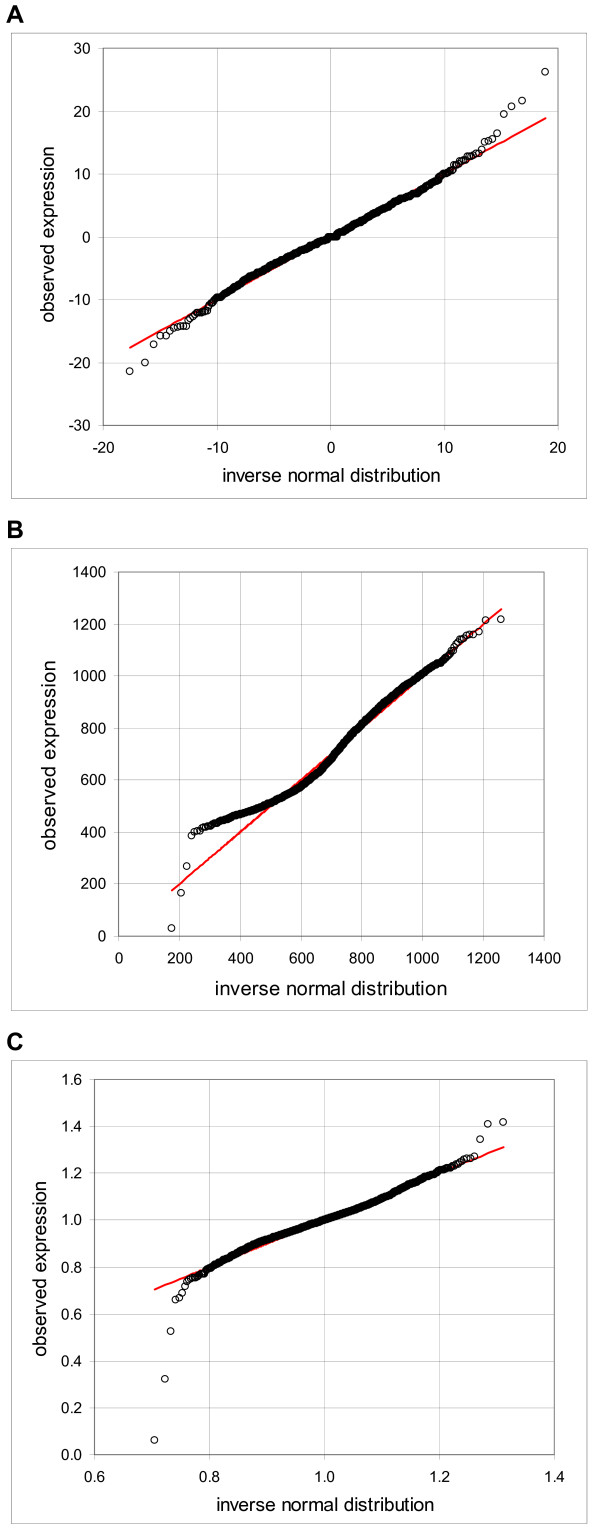
**Comparison of the observed frequency distribution to the inverse normal cumulative distribution, pooled data**. Quantile-quantile plots show on y-axis the observed expression and on x-axis value of the corresponding inverse normal cumulative distribution. Microarray data are derived from HuGeneFL, using the cell line IMR90 with 11 samples, pooled data. **A**: expression range from -0.1 to 0.1, 62 probe sets; **B**: expression range from 500 to 1000, 185 probe sets; **C**: expression range from 500 to 1000, 185 probe sets, relative expression values (sample expression divided by the mean of 11 samples; data Novak et al. [10]).

Furthermore, we observed that the probe sets with the mean expressions within a "reasonably small" range had, on average, a similar variance. Figure 2A shows pooled data of the 62 probe sets in the expression range from -0.1 to 0.1 (cell line IMR90, 11 replicates, Ref. [10]) in Q-Q plot in comparison to the inverse normal cumulative distribution with good agreement except for about six outliers. The picture changes when we scan probe sets with a wide range of mean expressions. Figure 2B shows the Q-Q plot of 185 probes sets in the range of means from 500 to 1000; the lower part of the graph deviates substantially from the straight line. When we plotted the relative expression (i.e. expressions of the individual probe sets divided by the mean of 11 arrays; Figure 2C), we got all the points, except for about ten outliers, back on the 45° line. This implies that the standard deviation is linearly proportional to the mean expression level.

**Figure 3:**
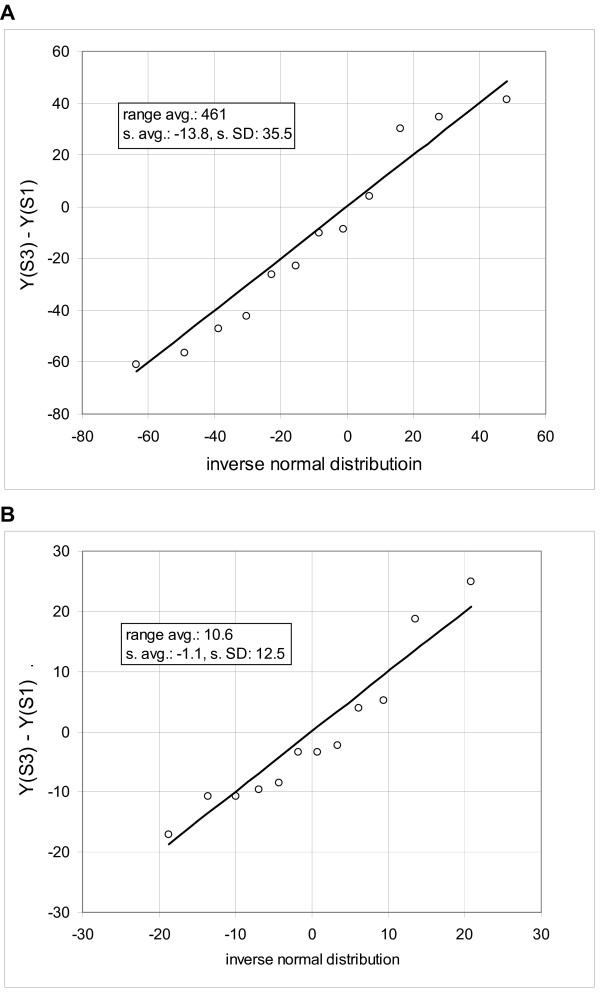
**Comparison of the observed frequency distribution of consecutive samples to the inverse normal cumulative distribution**. Quantile-quantile plots show on y-axis the difference of expression of two microarrays and on x-axis value of the corresponding inverse normal cumulative distribution. Microarray data are derived from HuGeneFL, using cell line IMR90 [10]. Probe sets of the microarrays 1 and 3 are ordered according to the mean expression and statistical samples of 12 probe sets are taken in the range of ranks from 250 to 4800. Panels show the samples with the Kolmogorov-Smirnov maximum distance equal or close to the mean value in the specified average expression rage. Inserts provide the average mean expression (range avg.), mean of the differences (s. avg.) and standard deviation (s. SD). **A**: expression range from 400 to 620, average D in the range is 0.142, sample D is 0.142; **B**: expression range from 10 to 20, average D in the range is 0.204, sample D is 0.204.

Based on the evidence of Figure 2, we hypothesize that approximately the same standard deviation can be obtained by scanning the data vertically, i.e. looking at expressions of the neighboring probe sets, or horizontally, i.e. looking at the series of arrays for each probe set. In other words, the probability that we will observe a difference *d *between the measurements *M*_1 _and *M*_2 _of the probe set *Pr*_1 _on the arrays *A*_1 _and *A*_2 _is, at least in the first approximation, about the same as the probability that we will observe such difference between the measurement *M*_3 _of the probe set *Pr*_1 _on the array *A*_1 _and the measurement *M*_4 _of the probe set *Pr*_2 _on the array *A*_2_, provided that the mean expression of both populations is the same. It further follows that an estimate of mean standard deviation of a group of genes with approximately same mean expression can be obtained from comparison of two arrays. We need to rank the probe sets according to the mean expression and evaluate the standard deviation from the differences in gene expressions in samples of *k *consecutive genes; the range of the means within a sample must be small. Furthermore, in this arrangement we can also obtain the standard deviation by using the ranked probe sets of each individual array (Ref. [10], Supplementary Material). Note that the standard deviation derived from the difference converges to √2σ, where σ is a standard deviation of a given population. Figure 3 shows a comparison of the frequency distribution of the difference in expression of two consecutive samples with the corresponding inverse normal cumulative distribution (cell line IMR90).

### Consecutive sampling analysis

**Figure 4:**
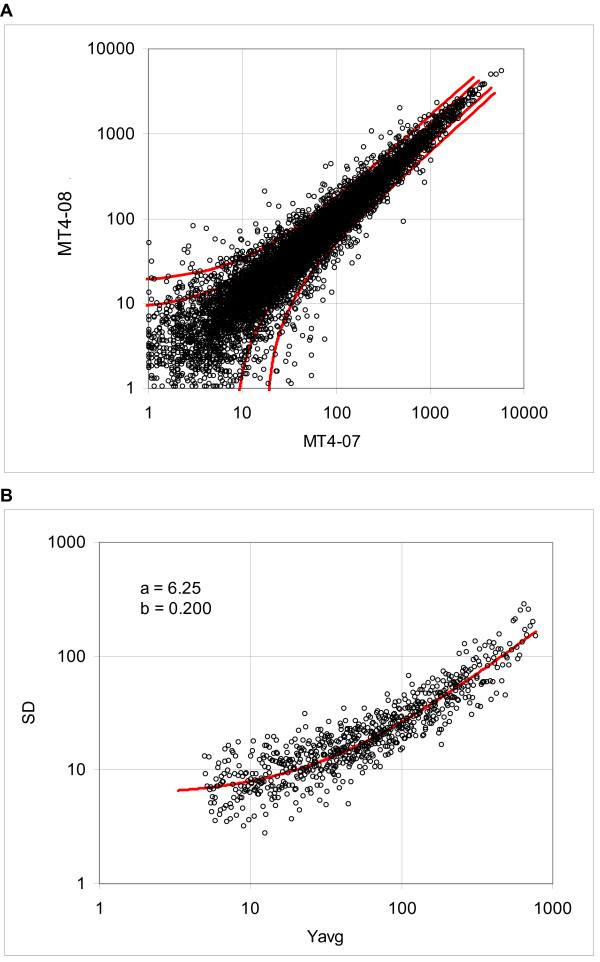
**Dispersion of the murine tissue data, array MG-U74Av2, samples MT4-07 and MT4-08**. **A**. Dispersion plot and boundaries of the 0.8 and 0.95 probability intervals. **B**: Standard deviations calculated using the expression difference in consecutive samples and the regression curve (solid line), representing the standard deviation function (data Cosio).

Assume, as a working hypothesis, that we can estimate the standard deviation of the gene expression variability of series replicate arrays from two-array comparisons. Since the evidence derived from the frequency distribution suggests that the standard deviation is linearly proportional to the expression level (at least in the first approximation), we assume that a representative estimate of the standard deviation can be obtained in the form of a linear function of the mean expression. A similar model was proposed on a basis of theoretical considerations by Rocke and coworkers [11-14]. The consecutive sampling program (see Methods) takes *k *pairs of expression values *Y*_*1i *_and *Y*_*2i *_ranked according to the mean (*Y*_*1i*_, *Y*_*2i*_) and calculates the standard deviation from the difference *Y*_*2i*_*-Y*_*1i*_, where the subscripts *1 *and *2 *denote the array number and *i *signifies the probe set rank; typically we set *k *= 12, 25 or 50, depending on the size of the array. The standard deviation function is then determined by fitting the logarithmically transformed values to the logarithm of the linear function of the mean expression (see the Methods section). For illustration, Figure 4A shows the dispersion plot and boundaries of the 0.8 and 0.95 probability intervals for the murine array MG U74Av2 (lung tissue, AKR mice; Table 4), whereas Figure 4B shows standard deviations of the consecutive samples consisting of 12 ordered pairs of probe sets and the regression curve, representing the standard deviation function.

**Table 4:** Summary of values of the coefficients of standard deviation function and *K*_*α *_coefficients

GeneChip/average/SD/CV	No. of probe sets	Cons. samp. size	No. of arrays	No. of comp.	Coefficient. of st. dev. function		Average Kalpha values in given probability intervals							
					
					a1	a2	0.500	0.600	0.700	0.800	0.900	0.950	0.990	0.995
***Total average [sum]***			***[682]***	***[900]***	***9.66***	***0.266***	***0.642***	***0.808***	***1.009***	***1.275***	***1.711***	***2.145***	***3.226***	***3.741***
***Total SD***					***9.99***	***0.146***	***0.033***	***0.039***	***0.043***	***0.045***	***0.052***	***0.080***	***0.270***	***0.390***
***Total CV***					***1.03***	***0.550***	***0.052***	***0.049***	***0.043***	***0.035***	***0.030***	***0.037***	***0.084***	***0.104***

HuGeneFL [a1]	7070	12	11	54	8.61	0.115	0.64	0.81	1.01	1.27	1.71	2.16	3.41	4.09
HuGeneFL [b]	7070	12	13	78	8.09	0.418	0.61	0.77	0.97	1.24	1.73	2.24	3.58	4.18
HuGeneFL [c]	7070	25	5	10	34.58	0.435	0.61	0.76	0.94	1.20	1.60	2.03	3.39	4.10
HuGeneFL [c]	7070	25	5	10	28.93	0.393	0.59	0.73	0.92	1.18	1.60	2.07	3.42	4.07

***HuGeneFL avg [sum]***			***[34]***	***[152]***	***20.05***	***0.34***	***0.61***	***0.77***	***0.96***	***1.22***	***1.66***	***2.13***	***3.45***	***4.11***
***HuGeneFL SD***					***13.71***	***0.15***	***0.02***	***0.03***	***0.04***	***0.04***	***0.07***	***0.10***	***0.09***	***0.05***
***HuGeneFL CV***					***0.68***	***0.44***	***0.04***	***0.04***	***0.04***	***0.04***	***0.04***	***0.05***	***0.03***	***0.01***

HG-U95Av2 [d]	12559	12	5	10	6.30	0.688	0.61	0.78	1.00	1.30	1.83	2.32	2.98	3.26
HG-U95Av2 [e1]	12559	25	15	15	10.49	0.199	0.65	0.82	1.01	1.27	1.68	2.07	3.05	3.55
HG-U95Av2 [e2]	12559	25	15	15	10.59	0.200	0.64	0.81	1.01	1.26	1.67	2.07	3.07	3.56
HG-U95Av2 [e3]	12559	25	15	15	10.41	0.189	0.63	0.80	1.00	1.25	1.66	2.07	3.06	3.55
HG-U95Av2 [e4]	12559	25	12	12	10.91	0.185	0.63	0.79	0.99	1.24	1.66	2.04	3.02	3.50
HG-U95Av2 [e5]	12559	25	4	2	6.50	0.394	0.65	0.82	1.01	1.26	1.65	2.05	3.05	3.62
HG-U95Av2 [f]	12559	25	4	2	5.64	0.155	0.64	0.81	1.00	1.25	1.66	2.04	2.92	3.31
HG-U95Av2 [g1]	12559	25	2	1	4.79	0.479	0.61	0.76	0.95	1.20	1.64	2.10	3.22	3.71
HG-U95Av2 [g1]	12559	25	5	10	3.31	0.500	0.64	0.80	1.00	1.26	1.69	2.09	2.98	3.29

***HG-U95Av2 avg [sum]***			***[77]***	***[82]***	***7.66***	***0.33***	***0.63***	***0.80***	***1.00***	***1.26***	***1.68***	***2.09***	***3.04***	***3.48***
***HG-U95Av2 SD***					***2.94***	***0.19***	***0.02***	***0.02***	***0.02***	***0.03***	***0.06***	***0.09***	***0.08***	***0.16***
***HG-U95Av2 CV***					***0.38***	***0.57***	***0.02***	***0.02***	***0.02***	***0.02***	***0.04***	***0.04***	***0.03***	***0.05***

HG-U95B [d]	12563	12	5	10	16.26	0.636	0.63	0.80	1.01	1.30	1.80	2.28	3.18	3.68
HG-U95B [e5]	12563	25	2	1	17.95	0.167	0.64	0.80	0.99	1.24	1.65	2.02	3.08	3.54
HG-U95C [d]	12587	12	5	10	20.57	0.603	0.64	0.81	1.02	1.30	1.77	2.25	3.39	3.98
HG-U95C [e5]	12587	25	2	1	16.42	0.178	0.63	0.79	0.99	1.23	1.62	2.00	3.06	3.61
HG-U95D [d]	12587	12	5	10	39.79	0.501	0.62	0.78	0.99	1.28	1.77	2.33	3.80	4.42
HG-U95D [e5]	12587	25	2	1	54.45	0.240	0.63	0.79	0.99	1.23	1.62	2.05	2.98	3.48
HG-U95E [d]	12582	12	5	10	31.84	0.534	0.60	0.76	0.97	1.27	1.78	2.29	3.53	4.08
HG-U95E [e5]	12582	25	2	1	45.64	0.215	0.63	0.79	1.00	1.25	1.64	2.02	2.88	3.31

***HG-U95B to E avg [sum]***			***[28]***	***[44]***	***30.37***	***0.38***	***0.63***	***0.79***	***0.99***	***1.26***	***1.71***	***2.16***	***3.24***	***3.76***
***HG-U95B to E SD***					***14.87***	***0.20***	***0.01***	***0.01***	***0.02***	***0.03***	***0.08***	***0.14***	***0.31***	***0.37***
***HG-U95B to E CV***					***0.49***	***0.53***	***0.02***	***0.02***	***0.02***	***0.02***	***0.05***	***0.07***	***0.10***	***0.10***

HG-U133A 2.0 [e6]	22225	25	15	15	4.55	0.091	0.65	0.82	1.02	1.28	1.72	2.14	3.14	3.62
HG-U133A 2.0 [e7]	22225	25	15	15	4.50	0.106	0.66	0.84	1.04	1.31	1.75	2.17	3.18	3.63
HG-U133A 2.0 [e8]	22225	25	12	12	4.10	0.108	0.67	0.84	1.05	1.32	1.77	2.21	3.26	3.75
HG-U133A 2.0 [h1]	22225	25	8	4	3.91	0.288	0.64	0.80	1.00	1.26	1.67	2.08	3.05	3.54
HG-U133A 2.0 [i1]	22225	12	4	6	6.25	0.210	0.65	0.82	1.01	1.27	1.68	2.08	3.04	3.48
HG-U133A 2.0 [j]	22225	25	5	10	6.54	0.390	0.62	0.79	0.98	1.25	1.68	2.10	3.13	3.62
HG-U133A 2.0 [j]	22225	25	5	10	5.54	0.393	0.62	0.79	0.98	1.25	1.66	2.08	3.10	3.64
HG-U133A 2.0 [k1]	22225	25	6	15	3.68	0.672	0.63	0.80	1.00	1.27	1.71	2.11	2.84	3.16
HG-U133A 2.0 [k1]	22225	25	3	3	4.95	0.435	0.59	0.74	0.93	1.19	1.65	2.15	3.37	3.79
HG-U133A 2.0 [l1]	22225	25	6	3	6.45	0.151	0.64	0.81	1.01	1.27	1.68	2.08	3.06	3.55
HG-U133A 2.0 [l2]	22225	25	12	6	6.78	0.148	0.65	0.82	1.02	1.27	1.67	2.06	2.93	3.33

***HG-U133A 2.0 avg [sum]***			***[91]***	***[99]***	***5.21***	***0.27***	***0.64***	***0.80***	***1.00***	***1.27***	***1.69***	***2.11***	***3.10***	***3.56***
***HG-U133A 2.0 SD***					***1.15***	***0.18***	***0.02***	***0.03***	***0.03***	***0.03***	***0.04***	***0.05***	***0.15***	***0.18***
***HG-U133A 2.0 CV***					***0.22***	***0.67***	***0.03***	***0.03***	***0.03***	***0.03***	***0.02***	***0.02***	***0.05***	***0.05***

HG-U133 Plus 2 [i2]	54000	50	8	10	3.94	0.188	0.68	0.85	1.06	1.33	1.76	2.17	3.11	3.55
HG-U133 Plus 2 [l3]	54000	50	20	27	4.03	0.086	0.65	0.82	1.02	1.28	1.69	2.07	2.95	3.34

***HG-U133 Plus 2 avg [sum]***			***[28]***	***[37]***	***3.99***	***0.14***	***0.67***	***0.83***	***1.04***	***1.30***	***1.72***	***2.12***	***3.03***	***3.44***

HG-Focus [k2]	8756	12	9	36	4.13	0.216	0.70	0.87	1.08	1.35	1.76	2.15	3.06	3.47
HG-Focus [k2]	8756	12	4	6	4.13	0.181	0.68	0.86	1.07	1.33	1.76	2.18	3.08	3.56
HG-Focus [k2]	8756	12	4	6	3.90	0.198	0.70	0.87	1.08	1.36	1.81	2.22	3.15	3.55
HG-Focus [k2]	8756	12	4	6	3.69	0.176	0.68	0.86	1.07	1.35	1.79	2.19	3.17	3.64
HG-Focus [k2]	8756	12	5	10	3.46	0.183	0.67	0.85	1.05	1.33	1.77	2.19	3.12	3.49
HG-Focus [k2]	8756	12	4	6	4.01	0.205	0.71	0.89	1.10	1.38	1.81	2.19	3.11	3.53
HG-Focus [k2]	8756	12	4	6	3.98	0.174	0.68	0.85	1.06	1.32	1.74	2.14	3.06	3.43

***HG-Focus avg [sum]***			***[34]***	***[76]***	***3.90***	***0.19***	***0.69***	***0.86***	***1.07***	***1.35***	***1.78***	***2.18***	***3.11***	***3.52***
***HG-Focus SD***					***0.24***	***0.02***	***0.01***	***0.02***	***0.02***	***0.02***	***0.03***	***0.03***	***0.04***	***0.07***
***HG-Focus CV***					***0.06***	***0.08***	***0.02***	***0.02***	***0.01***	***0.01***	***0.01***	***0.01***	***0.01***	***0.02***

MG-Mu11kSubA, SubB [a]	13069	12	10	20	9.98	0.121	0.59	0.75	0.95	1.22	1.70	2.20	3.65	4.48
MG-Mu11kSubA, SubB [a]	13069	12	10	20	8.03	0.170	0.59	0.74	0.94	1.20	1.68	2.19	3.78	4.66
MG-Mu11kSubA, SubB [a]	13069	12	10	20	8.21	0.145	0.60	0.76	0.96	1.23	1.70	2.21	3.70	4.52
MG-Mu11kSubA, SubB [a]	13069	12	10	20	5.32	0.139	0.56	0.71	0.91	1.19	1.71	2.30	4.03	4.84
MG-Mu11kSubA, SubB [m1]	13069	12	8	4	13.86	0.321	0.64	0.81	1.01	1.28	1.73	2.22	3.62	4.24
MG Mu11kSubA, SubB [n1]	13069	12	20	10	11.84	0.420	0.66	0.82	1.01	1.26	1.71	2.21	3.61	4.32

***MG-Mu11kSubA, SubB avg [sum]***			***[68]***	***[94]***	***9.54***	***0.219***	***0.61***	***0.77***	***0.96***	***1.23***	***1.70***	***2.22***	***3.73***	***4.51***
***MG-Mu11kSubA, SubB SD***					***3.03***	***0.122***	***0.04***	***0.04***	***0.04***	***0.04***	***0.02***	***0.04***	***0.16***	***0.22***
***MG-Mu11kSubA, SubB CV***					***0.32***	***0.557***	***0.06***	***0.05***	***0.04***	***0.03***	***0.01***	***0.02***	***0.04***	***0.05***

Mu19kSubA, B, C [m2]	12420	12	12	6	15.41	0.314	0.63	0.79	0.99	1.25	1.73	2.26	3.95	4.56

MG-U74Av2 [o]	12588	12	6	6	8.72	0.180	0.59	0.75	0.95	1.23	1.70	2.20	3.56	4.30
MG-U74Av2 [o]	12588	12	5	4	6.97	0.230	0.58	0.74	0.94	1.23	1.71	2.24	3.68	4.48
MG-U74Av2 [p]	12588	12	7	21	9.50	0.125	0.59	0.75	0.95	1.24	1.72	2.21	3.54	4.30
MG-U74Av2 [q]	12588	12	5	4	4.97	0.229	0.68	0.85	1.06	1.33	1.76	2.19	3.23	3.76
MG-U74Av2 [l4]	12588	12	9	9	7.50	0.111	0.67	0.83	1.03	1.30	1.73	2.13	3.09	3.53
MG-U74Av2 [r]	12588	12	10	20	4.69	0.269	0.65	0.82	1.02	1.29	1.73	2.17	3.31	3.89
MG-U74Av2 [r]	12588	12	3	3	3.30	0.238	0.64	0.80	1.00	1.27	1.71	2.17	3.48	4.04
MG-U74Av2 [e5]	12588	12	2	1	3.83	0.184	0.64	0.81	1.00	1.27	1.69	2.10	3.15	3.80
MG-U74Av2 [g2]	12588	12	2	1	13.50	0.451	0.64	0.81	1.01	1.27	1.72	2.16	3.36	3.95
MG U74Av2 [n2]	12400	12	26	13	6.56	0.113	0.64	0.80	1.00	1.27	1.70	2.12	3.15	3.67

***MG-U74Av2 avg [sum]***			***[75]***	***[82]***	***6.96***	***0.213***	***0.63***	***0.80***	***1.00***	***1.27***	***1.72***	***2.17***	***3.36***	***3.97***
***MG-U74Av2 SD***					***3.07***	***0.101***	***0.03***	***0.04***	***0.04***	***0.03***	***0.02***	***0.04***	***0.20***	***0.31***
***MG-U74Av2 CV***					***0.44***	***0.472***	***0.06***	***0.05***	***0.04***	***0.02***	***0.01***	***0.02***	***0.06***	***0.08***

MG-U430A [l5]	22636	25	10	5	7.68	0.132	0.65	0.81	1.01	1.27	1.68	2.06	2.94	3.36
MG-U430A [l6]	22636	25	5	10	10.08	0.265	0.67	0.84	1.04	1.30	1.71	2.10	2.98	3.35
MG-U430A [l6]	22636	25	5	10	9.44	0.160	0.65	0.81	1.01	1.27	1.67	2.05	2.93	3.30

***MG-U430A***			***[20]***	***[25]***	***9.07***	***0.186***	***0.65***	***0.82***	***1.02***	***1.28***	***1.69***	***2.07***	***2.95***	***3.34***
***MG-U430A***					***1.24***	***0.070***	***0.01***	***0.02***	***0.02***	***0.02***	***0.02***	***0.03***	***0.03***	***0.03***
***MG-U430A***					***0.14***	***0.377***	***0.02***	***0.02***	***0.02***	***0.02***	***0.01***	***0.01***	***0.01***	***0.01***

RG-U34A [h2]	8740	12	35	34	1.82	0.316	0.64	0.81	1.01	1.28	1.73	2.21	3.40	3.95
RG-U34A [l7]	8740	12	6	3	3.25	0.226	0.68	0.85	1.06	1.33	1.76	2.17	3.20	3.77
RG-U34A, [l8]	8740	12	4	2	6.01	0.146	0.65	0.83	1.03	1.29	1.72	2.11	3.10	3.60

***RG-U34A avg [sum]***			***[45]***	***[39]***	***3.70***	***0.229***	***0.66***	***0.83***	***1.03***	***1.30***	***1.74***	***2.16***	***3.24***	***3.78***
***RG-U34A SD***					***2.13***	***0.085***	***0.02***	***0.02***	***0.02***	***0.02***	***0.02***	***0.05***	***0.15***	***0.17***
***RG-U34A CV***					***0.58***	***0.371***	***0.03***	***0.03***	***0.02***	***0.02***	***0.01***	***0.02***	***0.05***	***0.05***

***RT-U34 Neurobiology [l7]***	***982***	***12***	***40***	***20***	***1.77***	***0.194***	***0.60***	***0.75***	***0.96***	***1.22***	***1.65***	***2.08***	***3.12***	***3.38***

***Drosophila [s]***	***13976***	***12***	***6***	***6***	***2.20***	***0.081***	***0.66***	***0.83***	***1.04***	***1.31***	***1.73***	***2.17***	***3.21***	***3.67***

E. coli [t]	7290	12	38	39	3.09	0.337	0.65	0.83	1.04	1.32	1.78	2.24	3.45	4.03
E. coli [u]	7290	12	15	30	1.88	0.302	0.65	0.83	1.04	1.34	1.81	2.29	3.47	4.04

***E. Coli avg [sum]***			***[53]***	***[69]***	***2.23***	***0.228***	***0.64***	***0.81***	***1.02***	***1.30***	***1.74***	***2.19***	***3.31***	***3.78***

ATH1 [v1]	22700	25	14	17	9.22	0.307	0.68	0.85	1.05	1.31	1.71	2.09	2.95	3.31
ATH1 [v1]	22700	25	34	36	11.18	0.269	0.68	0.85	1.05	1.31	1.71	2.08	2.91	3.26
ATH1 [w]	22700	25	8	4	6.26	0.279	0.65	0.81	1.01	1.27	1.69	2.10	3.06	3.45
ATH1 [x]	22700	25	4	2	3.09	0.232	0.68	0.85	1.05	1.31	1.72	2.12	3.17	3.70
ATH1 [y]	22700	25	4	2	3.07	0.247	0.66	0.82	1.02	1.28	1.68	2.08	3.09	3.60

***ATH1 avg [sum]***			***[64]***	***[61]***	***6.57***	***0.267***	***0.67***	***0.84***	***1.04***	***1.30***	***1.70***	***2.09***	***3.03***	***3.46***
***ATH1 SD***					***3.63***	***0.029***	***0.01***	***0.02***	***0.02***	***0.02***	***0.02***	***0.02***	***0.11***	***0.19***
***ATH1 CV***					***0.55***	***0.108***	***0.02***	***0.02***	***0.02***	***0.02***	***0.01***	***0.01***	***0.03***	***0.05***

Arabidopsis [v2]	8200	12	7	8	7.69	0.403	0.75	0.94	1.14	1.40	1.79	2.15	2.89	3.18

No. of probe sets is approximate number of the probe sets on array, Cons. samp. size is number of the probe pairs in a consecutive sample, No. of arrays is a number of arrays tested, No. of comp. is the number of pair-wise comparisons among the replicates, coefficients *a*_1 _and *a*_2 _are the coefficients of standard deviation function and *Kalpha *is the coefficient determining probability interval; "h." stands for "human," "m." for "murine." Average values, sum of arrays and comparisons (in square brackets), standard deviations (SD) and coefficients of variation (CV) of each GeneChip type are printed in bold italics.

Data sources:

a1 – J. P. Novak et al., HuGeneFl, IMR90 human cell line [10].

a2 – J. P. Novak et al., HuGeneFl, mouse tissues, adult male C57BL/6 [10].

b – A.-M. Mes-Masson, P. Tonin and coworkers, HuGeneFL, normal ovarian surface epithelial (NOSE) primary cell cultures, private communication.

c – P. Permana, HuGeneFL, human skeletal muscle tissue [35].

d – P. Tonin and A.-M. Mes-Masson and coworkers, HG-U95A to E, epithelial ovarian cancer (EOC) cell line [36].

e1 – Affymetrix, HG-U95A, latin square, experiments 1 to 5.

e2 – Affymetrix, HG-U95A, latin square, experiments 6 to 10.

e3 – Affymetrix, HG-U95A, latin square experiments 11 to 15.

e4 – Affymetrix, HG-U95A, latin square, experiments 16 to 19.

e5 – Affymetrix, HG-U95A to E, Demo Data.

e6 – Affymetrix, HG-U133A, latin square, experiments 1 to 5.

e7 – Affymetrix, HG-U133A, latin square, experiments 6 to 10.

e8 – Affymetrix, HG-U133A, latin square, experiments 11 to 14.

f – M. S. Rolph, HG-U95Av2, primary human bronchial epithelial cells.

g1 – Z. Gatalica, HG-U95Av2, breast tumor tissues and normal breast tissue samples [37].

g2 – Z. Gatalica, MG-U74Av2, mouse kidney tissue.

h1 – M. Boerma, HG-U133A 2.0, primary human umbilical vein endothelial cells (HUVECs) and the immortalized HUVEC cell line EA.hy926 [38].

h2 – M. Boerma, RG-U34A, cultures enriched for neonatal rat cardiac myocytes or fibroblasts [39].

i1 – M. Hajduch, HG-U133A 2.0.

i2 – M. Hajduch, HG-U133 plus 2.

j – S. Y. Kim and D. J. Volsky, HG-U133A 2.0, human fetal astrocytes, normal and pseudotyped HIV-1 infected [40].

k1 – O. Modlich, HG-U133A 2.0, human superficial and invasive bladder tumors [41].

k2 – O. Modlich and Raschke, Focus arrays, human lymphoma cell line Kaspas-422, DSMZ no.: ACC 32 (follicular B cell).

l1 – C. Wang and J. Xu, HG-U133A 2.0, human lymphoblast cell line.

l2 – C. Wang and J. Xu, HG-U133A 2.0, human pancreatic islet.

l3 – C. Wang and J. Xu, HG-U133 Plus 2, Stratagene Universal Human Reference RNA, Ambion Human Brain Reference RNA and mixtures of both in different concentrations.

l4 – C. Wang and J. Xu, MG-U74Av2, mouse biliary epithelial cells.

l5 – C. Wang and J. Xu, MG-U430A, mouse spleen.

l6 – C. Wang and J. Xu, MG-U430A, myofibroblast cell line.

l7 – C. Wang and J. Xu, RG-U34A, rat livers.

l8 – C. Wang and J. Xu, RG-U34A, rat bone marrow stem cells.

m1 – McInnes and coworkers, MG-Mu11kSubA, SubB, retinal RNA samples from WT and Rom1 knock-out mice [36].

m2 – McInnes, Szego and coworkers, MG-Mu19kSubA, SubB, SubC, retinal RNA samples from WT and Rom1 knock-out mice [42].

n1 – Burton, McGehee and coworkers, MG-Mu11kSubA, SubB, 3T3-L1 adipocytes [43].

n2 – Burton, McGehee and coworkers, MG-U74Av2,3T3-L1 adipocyte cultures [44].

o – M. Cosio, MG-U74Av2, lung tissues, murine strains NZW and AKR.

p – R. St-Arnaud, MG-U74Av2, C2C12 cells [45].

q – J. Hidalgo, MG-U74Av2, cortex samples, C57B6 normal and IL6 KO mice [46].

r – D. Radzioch, C. Guilbault and coworkers, MG-U74Av2, C57BL/6 (B6) WT and C57BL/6-*Cftr*-/- (KO) inbred mice [15].

s – S. E. Choe, Drosophila, Drosophila Gene Collection release 1.0 cDNA clones [19].

t – F. Blattner, E. coli antisense genome, E. coli K-12 strain MG1655 and an isogenic fnr::Sp^r ^Sm^r ^strain [47].

u – J. Slonczewski and S. BonDurant, E. coli antisense genome, E. coli K-12 strain W3110 [48].

v1 – E. Blumwald and J. Sottosanto, ATH1, A. thaliana ecotype Wassilewskija, wild-type line (WS), nhx1 'knockout' line, and a knockout restoration line; [49] and unpublished data.

v2 – E. Blumwald and J. Sottosanto, arabidopsis, A. thaliana ecotype Wassilewskija, wild-type line (WS), and a nhx1 'knockout' line (unpublished data).

w – D. Honys and D. Twell, ATH1, Arabidopsis thaliana ecotype Landsberg erecta plants [50].

x – E. Nambara and K. Nakabayashi, ATH1, Arabidopsis thaliana (L.) Heynh of ecotype Columbia [51].

y – E. Nambara and K. Tatematsu, ATH1, Arabidopsis thaliana (L.) Heynh of ecotype Columbia [52].

**Figure 5:**
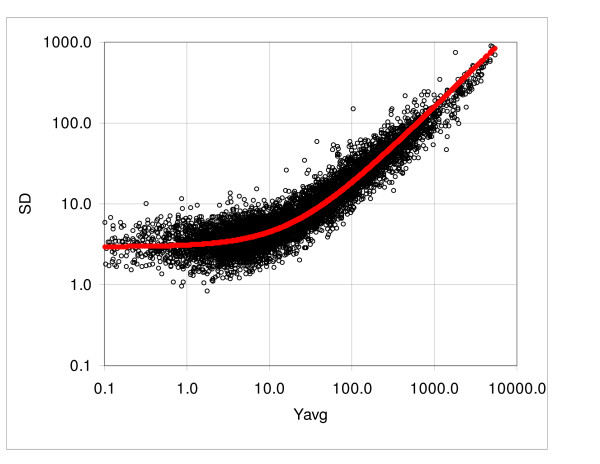
**Standard deviation of the Focus arrays, arrays 01 to 09**. Standard deviations are calculated from the individual probe sets of nine samples. The solid curve represents the standard deviation function derived from the consecutive sampling. The regression curve corresponding to logarithm of the linear standard deviation function fitted to logarithm of the experimental standard deviation (not shown) overlaps the consecutive sampling approximation; the coefficients obtained from consecutive sampling are *a*_1 _= 2.92√2 and *a*_2 _= 0.153√2 and the regression coefficients obtained from individual probe sets are *a*_1 _= 2.87 and *a*_2 _= 0.154 (data Modlich, Focus 1).

To verify our working hypothesis stated above, we also evaluated the regression function using the standard deviations calculated from dispersion of expressions recorded by replicates of the individual probe sets. Table 3 shows the comparison for five assays with the number of replicates ranging from four to 11. The values of the coefficients *a*_1 _and *a*_2 _ranged from 2.1 to 6.0 and from 0.076 to 0.161, respectively. Since the standard deviation calculated from the difference is √2 times larger than the standard deviation of a given population, we compared the values obtained from the individual genes to the results of the consecutive sampling divided by √2. The average difference of the coefficient *a*_1 _for the Affymetrix arrays was 5.4% and for the Illumina arrays 7.4%, whereas the differences for the coefficient *a*_2 _were 7.5% and 11.2%, respectively. The total average difference for *a*_1 _and *a*_2 _was 6.2% and 9.0%, respectively. We observed that in all cases except one (Focus Arrays) the values obtained from the consecutive sampling were above the results obtained from individual genes. This is to be expected, because the expressions in the consecutive samples belong to populations with different, albeit very similar, means. Since the standard deviation increases with increasing average, the differences among the means introduce an additional variability. Figure 5 shows an example of the standard deviation derived from 9 replicates of the Focus array. The points represent the standard deviations of the expressions of individual probe sets and the solid line represents the standard deviation function derived from the consecutive sampling.

### Probability intervals and correlation of the *K*_*α *_coefficients with *t*-distribution

Once we evaluate the standard deviation function, we can determine the limits of the probability intervals, i.e. the boundaries corresponding to a distance from the 45° axis of symmetry equal to a constant number of standard deviations. Equations defining these limits are given in the Methods section (Eqs. (2) and (3)). The coefficient *K*_*α *_is equivalent to the standardized or "standard" deviate of the normal distribution, representing the distance from the mean, expressed in standard deviations. In case of the z-distribution or *t-*distribution the standard deviates corresponding to specific probability intervals can be derived from the cumulative distribution function. Since the theoretical distribution function corresponding to the probability intervals of the microarray dispersion is unknown, we determined the coefficients *K*_*α *_empirically. First we calculated the standard deviation function and then used Eqs. (2) and (3) to define the limits of the standard deviate intervals ("probability intervals"; see Figure 4A, note that the boundary lines appear in the log-log plot as curves). To determine the *K*_*α *_coefficients corresponding to specific probabilities we counted the points lying outside a given interval. For example, if the number of points in a given expression range examined was, say, 10000, we determined the *K*_*α *_value corresponding to the interval 0.995 by finding the interval containing 9950 points (99.5%), leaving the 50 points outside. More precisely, the *K*_*α *_is calculated as the average of the values corresponding to the integers above and below the number equal to the given fraction.

The *K*_*α *_coefficients are standardized with respect to the mean and standard deviation of given populations. As such, they are a universal measure of the probability of occurrence, function only of the shape of the distribution function. Considering the complexity of the processes involved in microarray experiments, we did not expect that the coefficient would be constant even for just a variety of RNA samples of a given type of array. Nonetheless, examination of 42 microarray studies with two to 11 replicates comprising 682 arrays and 22 Affymetrix array types revealed that values of the *K*_*α *_coefficients were very close for all tested comparisons (note that multiple chip arrays are counted as multiple types). The coefficients were invariant for a wide range of dispersions, invariant with respect to different laboratory conditions, different tissues and different species and across all the types of arrays we tested. Table 4 shows a summary of the average values of *K*_*α *_coefficients for 900 pair-wise comparisons. The average coefficient *a*_1 _varied from 1.8 to 54.4 and coefficient *a*_2 _from 0.08 to 0.69, with total coefficients of variation 1.05 and 0.54, respectively. In spite of such a wide range, the differences in the coefficient *K*_*α *_were small: the coefficient of variation ranged from the minimum 0.031 at the probability p = 0.9 to the maximum 0.101 at p = 0.995.

**Figure 6:**
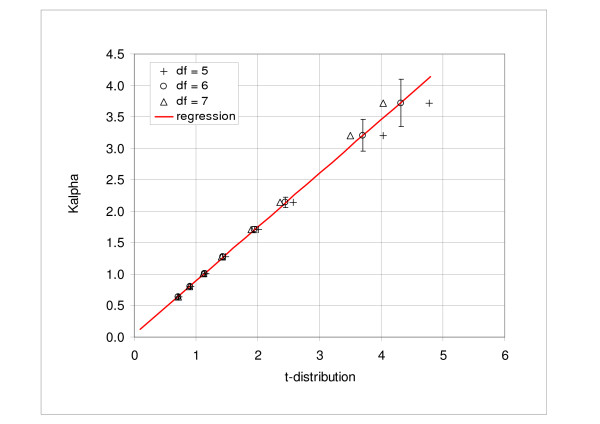
**Correlation of the *K*_*α *_coefficients and inverse *t*-distribution**. Figure shows the values of *K*_*α *_coefficient correlated with the corresponding values of the *t*-distribution in the range of probabilities from 0.5 to 0.995. The adjusted R^2 ^coefficient is 0.99993, intercept is 0.039 and the coefficient of proportionality is 0.855. The degree of freedom for the *t*-distribution is 6.

**Figure 7:**
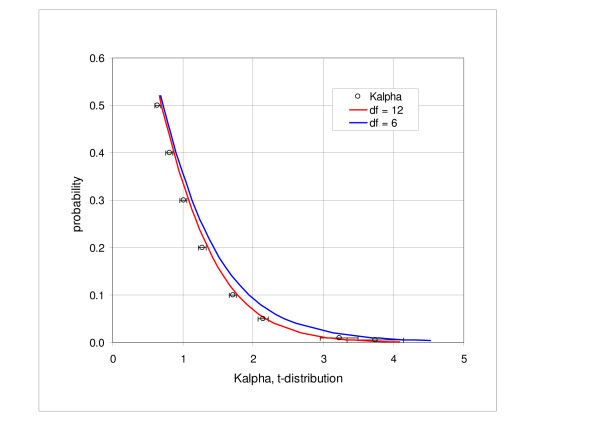
**Comparison of the *K*_*α *_distribution and inverse *t*-distribution**. *K*_*α *_values correspond to probabilities from 0.5 to 0.995. The degree of freedom for the inverse *t*-distribution (solid lines) is 6 and 12.

We examined the relationship between the coefficients *K*_*α *_and the inverse cumulative *t-*distribution. We found a very close linear correlation between the *K*_*α *_values and the *t-*distribution values corresponding to the degree of freedom df = 6. The adjusted R^2 ^coefficient was 0.99993, with the intercept of 0.039 and the coefficient of proportionality of 0.855. Figure 6 shows the graph of the *K*_*α *_values plotted against the *t-*distribution parameters in the range of probability intervals from 0.5 to 0.995; the solid line represents the regression line for df = 6 and the bars indicate the standard deviation. We also compared directly the *K*_*α *_intervals and *t-*distribution. Figure 7 shows the probability values corresponding to the *K*_*α *_coefficients and *t-*distribution probability, represented by the solid curve. In the direct comparison we obtain better agreement for df = 12 than for df = 6.

**Figure 8:**
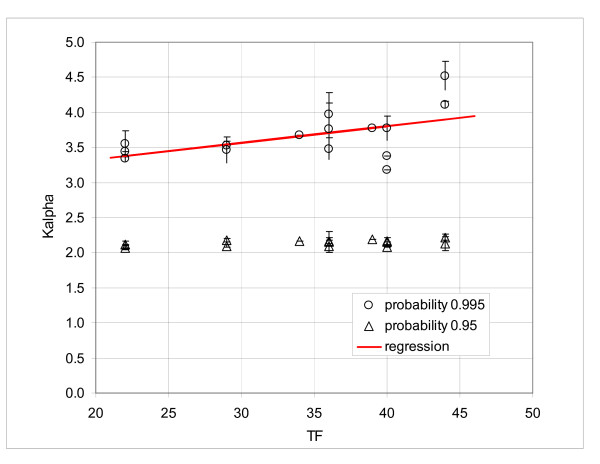
**Average *K*_*α *_coefficients at the intervals 0.95 and 0.995**. Correlation of the *K*_*α *_coefficients with the sum of the feature size and number of probe pairs; bars show the standard deviation for the interval 0.995.

A further examination of the results shown in Table 4 seemed to indicate that the older GeneChips^® ^had a somewhat broader distribution. For example, the mean *K*_*α *_at 0.995 for the array HuGene FL was 4.11, while these values for the later versions HG-U95A and HG-U133A were 3.48 and 3.56, respectively. To assess the correlation between the developing technology and shape of the *K*_*α *_distribution, we need a quantitative parameter, reflecting the technological advancement. One possibility is the feature size and number of probe pairs per set, which have been systematically decreasing with time. Table 5 shows the overview of the selected *K*_*α *_values correlated with the technical factor TF, defined as the sum of the feature size and number of the probe pairs per probe set. In Figure 8 we present the *K*_*α *_values at 0.95 and 0.995, plotted against TF. The regression line showed a slight decreasing tendency of the *K*_*α *_values at 0.995 with the decreasing TF, but the graph was not very convincing; the adjusted R^2 ^was only 0.31. No trend was discernible at the probability of 0.95.

**Table 5:** Overview of the GeneChip types

GeneChip	Feature size	Probe pairs	TF	No. of labs.	No. of arrays	Ka 0.95		Ka 0.99		Ka 0.995	
						
						avg	SD	avg	SD	avg	SD
HuGeneFL	24	20	44	2	34	2.13	0.10	3.45	0.09	4.11	0.05
HG-U95Av2	20	16	36	4	77	2.09	0.09	3.04	0.08	3.48	0.16
HG-U95B to E	20	16	36	2	28	2.16	0.14	3.24	0.31	3.76	0.37
HG-U133A 2.0	11	11	22	6	91	2.11	0.05	3.10	0.15	3.56	0.18
HG-U133 Plus 2	11	11	22	2	28	2.12	---	3.03	---	3.44	---
HG-Focus	18	11	29	1	34	2.18	0.03	3.11	0.04	3.52	0.07
MG-Mu11kSubA, SubB	24	20	44	2	80	2.22	0.04	3.73	0.16	4.52	0.20
Mu19kSubA, B, C	24	20	44	1	12	2.26	---	3.95	---	4.56	---
MG-U74Av2	20	16	36	6	75	2.17	0.04	3.36	0.20	3.97	0.31
MG-U430A	11	11	22	1	20	2.07	0.03	2.95	0.03	3.34	0.03
RG-U34A	24	16	40	2	45	2.16	0.05	3.24	0.15	3.78	0.17
RT-U34 Neurobiology	24	16	40	1	40	2.08	---	3.12	---	3.38	---
Drosophila	20	14	34	1	6	2.17	---	3.21	---	3.67	---
E. Coli	24	15	39	2	53	2.19	---	3.31	---	3.78	---
ATH1	18	11	29	4	64	2.09	0.02	3.03	0.11	3.46	0.19
Arabidopis [s2]	24	16	40	1	7	2.15	---	2.89	---	3.18	---

The first two columns of data show the feature size and number of the probe pairs per probe set. TF is the technical factor defined as the sum of feature size and probe pairs. No. of lab gives the number of different laboratories, where the data were generated. No. of arrays gives the number of arrays per the GeneChip type. The last three columns give the mean *K*_*α *_values at the probability 0.95, 0.99 and 0.995.

We found the probability intervals useful for estimating the significance of the observed differences, in particular in assays with small numbers of replicates (four or less). The *K*_*α *_coefficients representing the number of standard deviates that separates the measured values from the reference mean values provide an objective measure of dissimilarity between the populations under consideration. For the single normal population the interpretation is straightforward. However, in case of the microarray data we deal with the multitude of populations and the theoretical *K*_*α *_function is unknown; our correlation results though indicate that a universal function, encompassing all GeneChip^® ^types, exists. We could use the *K*_*α *_values obtained from correlations instead of the theoretical values; however, the experience has shown that the results are not reliable. First, considering the large number of values on the arrays even small differences in the *K*_*α *_function translate into substantial differences in number of candidates. Second, quite frequently the unplanned differences between the samples cause deviations from the expected behavior and render comparison with the general function unsuitable. Therefore, in practice, we use the *K*_*α *_coefficients only for ranking.

To determine the best candidate genes differentially expressed, we search for the genes with the largest *K*_*α *_in all or most of the comparisons. We named this method "consecutive sampling and coincidence test." Briefly, we calculate the *K*_*α *_coefficients in all possible *N *pair-wise comparisons and select the probe sets with expressions beyond a given probability interval in at least *M *comparisons; the upper limit of probability of observing *f *false positives can be calculated theoretically, assuming random selection. Detailed discussion is beyond the scope of this study (a particular example of application to the analysis of five-replicate assay of murine lung tissue can be found in Ref. [15]). The main advantages of this approach are that: 1) it is a nonparametric method; 2) applicable to assays with small number of replicates (as small as two); 3) it examines all pair-wise comparisons and makes easy to identify and automatically flag problematic arrays; 4) the probability of false positives can be easily calculated from the binomial distributions or estimated by straightforward simulations [8]. Here, as a brief illustration of the consistency of this approach, Table 6a shows the analysis of five replicates of murine GeneChips MG-U74Av2, labeled as mg1 to mg5 (data Ref. [15]). The purpose is to examine consistency of the results of analysis of differential expression using the *t*-test, coincidence method and RMA. For the test, we defined five subsets: [mg1, 2, 3, 4], [mg1, 2, 3, 5], [mg1, 2, 4, 5], etc. and selected the candidate genes. The threshold of selection for the *t*-test was P = 0.01, for the coincidence 12 out of possible 16 cases, and for the RMA minimum fold difference 2. We selected the genes satisfying the given criteria for each subset and subsequently counted the common genes found in any two particular subsets. The mean values of all possible comparisons are shown in the fourth row of Table 6a. The values shown in the last row represent the ratio of the mean number of common genes relative to the mean number of the genes that passed the test for each subset (third row) in percent. In the case of the *t*-test, the average for the over- and under-expressed genes was 23 and 29 percent, respectively. By comparison, the coincidence test for the over-expressed genes yielded 75% and RMA 81%; in the case of the coincidence and RMA, the mean numbers of under-expressed genes were below ten and the comparisons were considered unreliable (data not shown). In only this example we used MAS 5 generated values. Table 6b shows the results of similar tests carried out using the Illumina fiberoptic bead-based oligonucleotide arrays. In this case the average percentages of agreement for the coincidence tests were 89.1, to compare to 48.2%, obtained for the *t*-test. A more detailed comparison under slightly different assumptions, which includes also the CyberT and Tusher's method, can be found in Ref. [8].

**Table 6:** Summary of the results of consistency tests

**a)**				
	*t*-test: P < 0.010		Coincidence	RMA

Above or Below	above	below	above	above
Mean of 4-sample test	58.2	72.0	29.4	40.4
Common to 2 sets (mean)	13.5	20.5	22.1	32.9
SD	2.3	2.8	3.4	6.0
Ratio %	23.2	28.5	75.2	81.4
**b)**				

	Coincidence, interval 0.9	Coincidence, interval 0.8	*t*-test P = 0.0016	

Mean of 3-samples test (7 of 9)	12.3	17.5	11.0	
Common to 2 sets (average)	10.2	16.7	5.3	
Ratio (%)	83.0	95.2	48.2	

**a) **The *t*-test, coincidence test and RMA on MG-U75Av2 array (five samples; data Ref. [15]). The data were subject to one-tail *t*-test at the level 0.01, coincidence test and RMA. The coincidence and RMA tests were not carried out for the cases below the interval, since the numbers of occurrences were too small. The means of positive cases in five four-sample tests are given. The means of genes common to any two trials are shown. Ratio of the means is given in percent. **b) **The *t*-test and coincidence test, Illumina (four samples; data Ref. [8]). The second and third column list the number of genes identified by the coincidence method for the interval 0.9 and 0.8, respectively. The last column shows the numbers of genes that satisfied the *t*-test. The first and second rows of data give the mean number of genes that passed three-sample sets and the mean of the genes passing concurrently in two particular tests, respectively.

## Discussion

In our practice we adopted the approach of Affymetrix, which estimates the background from 2% of the probes with the lowest signals, uses the MM probes for the estimate of the non-specific component and yields an estimate of an "absolute" value of the RNA abundance. We adhered to the Affymetrix philosophy in spite of popularity of the global fitting methods, such as dChip [3,4] and RMA [6,7], because it provide us with a representative expression values independently for each array, enables us to assess consistency of the observed values and detect irregularities and outliers. This is an important advantage, considering how frequently we detect "atypical" arrays among replicates. Furthermore, consistency checks have shown similar rates of coincidence for both RMA and coincidence testing (Table 6a).

Results of the published studies comparing various methods of analysis are inconsistent and do not provide a clear guidance for selection of the method. Irizarry et al. [7], e.g., reported better detection of differentially expressed genes by RMA as compared to the dChip [4] and Affymetrix "Average Difference" (MAS 4) and MAS5 methods. Similarly, Barash et al. rated RMA as the best of the three with dChip performing slightly better than MAS5 [16]. Shedden et al. [17] claim superior results for dChip and "trimmed mean" and inferior results for MAS5 and one version of RMA (GCRMA-EB); the other version of RMA (CGRMA-MLE; Wu Z, Irizarry R, Gentleman R, Murillo F, Spencer F., 2003, A Model Based Background Adjustment for Oligonucleotide Expression Arrays, Technical Report, John Hopkins University, Department of Biostatistics Working Papers, Baltimore, MD) produced mixed results (in trimmed mean the PM-MM differences are ordered, 20% of the highest and lowest values are deleted and the mean of the remaining probe pairs represents a measure of gene expression). Han et al. [18] compared the Affymetrix MAS 5, dChip using PM-MM and PM only input and RMA. In this study the PM only variant of dChip and RMA showed the best performance. The authors also noted that the coefficient of variation in replicate experiments in the case of MAS 5 increases with a decreasing mean signal, but remains approximately constant for PM only of dChip and RMA. Invariance of the coefficient of variation raises a certain concern: percentage of contribution of the non-specific signal increases with the decreasing concentration and one would expect that at low concentrations it would be harder to separate it from the specific component. Choe et al. [19] compared various combinations of the six steps in the differential expression analysis: background subtraction, probe-level normalization, PM adjustment (correction for the non-specific signal), expression summary (derivation of the representative gene expression from the multiple probe signals), probe set-level normalization and statistical evaluation. This was a particularly interesting comparative study, since their experimental design was much closer to real conditions than spiked sets of arrays used in other publications. The authors report that the combination of the MAS5 for background correction and PM adjustment, median Polish method or, marginally inferior, MAS5 for expression summary, loess for normalization and CyberT for statistical evaluation [20] yielded the best results. They also emphasized that, under their particular conditions, MM signals provided the best estimate of the non-specific component. Furthermore, they concluded that in the statistical evaluation it is important to account for variation of the standard deviation with the mean expression (see also [21]). They adopted the CyberT model proposed by Baldi and Lang [20], which uses consecutive samples to estimate the expression-dependent component of the standard deviation, similarly to our approach.

In the present analysis of the frequency distributions and properties of the *K*_*α *_we used the MAS 4 software, instead of MAS 5 or GCOS. The reason is that these more recent versions distort the frequency distribution and standard deviation function in the near-zero region. In the case of the Affymetrix arrays the estimate of additive signal, caused by nonspecific binding and other spurious phenomena, is based on the mismatch signal. The estimate of the "true" gene expression is then derived from the difference between perfect match (PM) and mismatch (MM). However, in such system the variability of **this difference **is a "true" measure of the absolute gene expression variability. Negative difference **does not mean that the gene expression is negative**, but simply that the MM signal is larger than PM. It is perfectly logical that in absence of a given RNA the MM signal would exceed PM in about 50% of cases. The frequency distribution of the PM – MM difference in the absence of a specific RNA is the best measure of the constant component of spurious signal, added to the "true signal" value. Such estimate cannot be derived from MAS 5 or GCOS data. Replacing the negative values resulting from the signals actually measured by the PM and MM probes by arbitrary numbers introduces inconsistency in the method of evaluation and leads to decrease of the standard deviation with decreasing signal level in near-zero region (unpublished observation). In the low expression region it also leads to a substantial increase in number of probe sets that deviate from the normal distribution [22]. Nevertheless, at the expression levels above about 50 (normalized to 100% of the mean) our observations and conclusions hold even for the data analyzed with MAS 5 or GCOS. Some methods of analysis, such as RMA and one variant of the dChip, avoid the negative values without introducing inconsistency in evaluation by using the PM values only.

In the preceding section, we demonstrated that the frequency distribution of the random and pseudo-random fluctuations of microarray data is predominantly normal. The normal frequency distribution is a useful property, allowing straightforward identification of outliers, a convenient quality check and simple characterization of the observed data. Normality of the error term is an important assumption of various global models used for the analysis of measured probe signals, such as dChip [3,4], RMA [5-7] and other approaches [23-25]. Among these only Pavelka et al. [25] demonstrated that the assumption is justified. Normality is also a necessary condition for application of the parametric methods. Here we observed that on average over 5% of samples deviate from the normal distribution (using the test threshold of 0.05). It is agreed that the *t*-test and ANOVA are rather robust with respect to normality (e.g. SigmaStat software [SPSS inc.] uses for ANOVA the threshold of 0.01), nonetheless the noted deviations call for caution when using parametric methods, in particular considering that every analysis involves multiple testing. Our conclusion differs from that of Gilles and Kipling [22], who studied normality of GeneChip data using a set of 59 Affymetrix HG-U95A microarrays with human pancreatic cRNA. The authors concluded that "...data provide strong support for the application of parametric tests to GeneChip data sets without the need for data transformation." However, Shapiro-Wilks test, applied to the MAS 4 evaluated data, detected 28% of probe sets deviating from normality at the level P < 0.05. The authors argued that the Shapiro-Wilks test is, perhaps, too sensitive, since the Q-Q plots of the observed and normal values show high correlation. In our opinion, correlation is not a reliable measure of normality. The correlation coefficient can be high in spite of a small number of outlying points that might sufficiently affect variance to lead to false positive conclusions. Gilles and Kipling also observed an excessively high percentage of deviations from normality at low expression levels in data evaluated using MAS 5 and deduced that the most likely reason is MAS 5 treatment of negative values.

The probability of any value in normally distributed populations can be expressed as a number of standard deviates. For example, expressing the difference between the mean of a given population and a particular measurement in standard deviates enables us to compare this difference to the standardized z-distribution and determine, among other things, the cumulative probability of occurrence. For example, the standard deviate of 3.09 corresponds to the cumulative probability of measurements in the tails of the distribution function P = 0.001, a conventional threshold for identifying outliers in small-size samples. In the case of microarrays, we do not have single standard deviate values but standard deviate functions, defined by the *K*_*α *_coefficients. Nonetheless, the same reasoning applies. The necessary and sufficient condition for "standardization" of microarray dispersion is that the *K*_*α *_coefficients must be invariant. Under such condition differences expressed in *K*_*α *_variable are universal, independent of the particular properties of RNA samples, type of array, etc. This is of a practical significance for comparative studies, such as studies comparing results obtained in different laboratories [26-28], different generations of the Affymetrix array [29,30] or in different species [31-34].

Analysis of significance in assays with less than five replicates always represents a problem. Parametric methods are not reliable in the case of small samples and the nonparametric Mann-Whitney test and ANOVA on ranks provide a very crude estimate for three or four samples and are not very reliable either. Before asserting the invariance of *K*_*α *_values, we used probability intervals in pair-wise case-control comparisons and selected as candidate genes the genes that fell outside a given interval in predetermined number of comparisons [8,15]. We refer to this method as the "consecutive sampling and coincidence test." A more appropriate approach would be to estimate *K*_*α *_coefficients representing the random variability from replicate arrays and apply the coincidence test to the initial sets of genes lying outside the intervals defined by these values.

Besides the significance estimates, we found that the probability intervals determined by *K*_*α *_coefficients are very useful for filtering out the random probe sets prior to the clustering analysis, in particular when hierarchical clustering or principal component analysis is employed. Another straightforward application is to select the relevant set of genes for pathway analysis. Finally, disproportionate *K*_*α *_coefficients indicate a problematic pair of arrays, usually with nonlinear behavior or large clusters of outlying genes.

## Conclusion

We provide evidence that the majority of microarray samples, typically between 85 and 95 percent, conform to a Gaussian distribution. Monitoring excessive number of consecutive samples that fail the Kolmogorov-Smirnov normality test is a useful method of quality control in automated analysis of gene expressions.

We used the consecutive sampling method to determine *K*_*α *_coefficients defining the probability intervals in pair-wise comparisons. Subsequently, we demonstrated that these coefficients are, in the first approximation, independent of the nature of sample, the laboratory conditions and the type of array. The *K*_*α *_coefficients within the range of probabilities from 0.5 to 0.995 correlate very well with *t-*distribution. Filtering out the genes with expressions within the probability intervals defined by *K*_*α *_coefficients can significantly enhance the performance for clustering methods, especially for hierarchical clustering and principal component analysis. Finally, selecting the genes that fall outside a specific probability interval in a specific number of pair-wise comparisons provides a convenient, nonparametric method for estimating the significance of observed differences, advantageous, in particular, in case of assays with a small number of replicates.

Our main objective in studying the invariant properties of *K*_*α *_distribution was to examine the arrays from many different experiments in different laboratories, rather than replicate assays, to verify technology or method of analysis. The fact that even under such diversity of data the *K*_*α *_distribution is so stable and so close to *t-*distributions implies that the Affymetrix technology provides "true" representation of quantitative phenomena, involved in measurement of the abundance of RNA in studied media. However, improving the precision and devising the most effective methods of evaluation still remain a challenge for future development.

## Methods

### Consecutive sampling program

The first version of the consecutive sampling method was published by Novak et al. [10]. Briefly, the program ranks the probe sets of two arrays under comparison (say array A_1 _and A_2_) according to the mean expression and defines the samples of *k *consecutive pairs of values ("consecutive samples"; typically *k *= 12, 25 or 50, depending on the size of the array). Then it calculates the standard deviation of samples from the difference of expressions and fits logarithm of the linear function

*SD *= *a*_1 _+ *a*_2_*Y*_*mean *_    (1)

to the logarithm of calculated values; here *Y*_*mean *_is the sample mean and *a*_1 _and *a*_2 _are the intercept and coefficient proportionality, respectively. The logarithmic transform prior to the regression is used solely to equalize the residua. Without the transform the high-expression residua greatly outweigh the low-end values and lead to an inaccurate approximation in the near-zero range. After the fitting the standard deviation function is inverse-transformed back to the original scale. Since the range of mean values within the samples must be small, we exclude an adequate number of the probe sets below the maximum expression, where the density of the probe sets per unit of expression is low (see the identity test below). This is necessary to avoid inaccurate values caused by large differences of the mean values within the samples. Once the standard deviation function is determined, it is assumed it can be extrapolated to the maximum expression. Table 1 illustrates the procedure. The column "Rank" shows the rank from the highest mean expression, columns "Sample" give the expression values of the arrays A_1 _and A_2_, "*Y*_2_*-Y*_1_" is the expressions difference, "(Y2+Y1)/2" is the expression mean, "Sample Mean" is the mean expression of the sample, "SD(Y2-Y1)" is the standard deviation obtained from the difference of expressions and "SD(Y1)+SD(Y2)" is the sum of the standard deviations calculated from the values Y1 and Y2, respectively. The first 250 probe sets were excluded from the regression procedure to ensure that the variation of the mean expression of two arrays in a given consecutive sample is small.

When the standard deviation function is determined, the program calculates the boundaries of chosen probability intervals as functions of the mean expression. The upper and lower limits in the dispersion plot *Y*_2 _versus *Y*_1 _are defined as

YU=Y1+Kα(a1+a2Y1/2)1−Kαa2/2     (2)
 MathType@MTEF@5@5@+=feaafiart1ev1aaatCvAUfKttLearuWrP9MDH5MBPbIqV92AaeXatLxBI9gBaebbnrfifHhDYfgasaacH8akY=wiFfYdH8Gipec8Eeeu0xXdbba9frFj0=OqFfea0dXdd9vqai=hGuQ8kuc9pgc9s8qqaq=dirpe0xb9q8qiLsFr0=vr0=vr0dc8meaabaqaciaacaGaaeqabaqabeGadaaakeaacqWGzbqwdaWgaaWcbaGaemyvaufabeaakiabg2da9maalaaabaGaemywaK1aaSbaaSqaaiabigdaXaqabaGccqGHRaWkcqWGlbWsdaWgaaWcbaacciGae8xSdegabeaakiabcIcaOiabdggaHnaaBaaaleaacqaIXaqmaeqaaOGaey4kaSIaemyyae2aaSbaaSqaaiabikdaYaqabaGccqWGzbqwdaWgaaWcbaGaeGymaedabeaakiabc+caViabikdaYiabcMcaPaqaaiabigdaXiabgkHiTiabdUealnaaBaaaleaacqWFXoqyaeqaaOGaemyyae2aaSbaaSqaaiabikdaYaqabaGccqGGVaWlcqaIYaGmaaGaaCzcaiaaxMaadaqadiqaaiabikdaYaGaayjkaiaawMcaaaaa@4F2D@

and

YL=Y1−Kα(a1+a2Y1/2)1+Kαa2/2,     (3)
 MathType@MTEF@5@5@+=feaafiart1ev1aaatCvAUfKttLearuWrP9MDH5MBPbIqV92AaeXatLxBI9gBaebbnrfifHhDYfgasaacH8akY=wiFfYdH8Gipec8Eeeu0xXdbba9frFj0=OqFfea0dXdd9vqai=hGuQ8kuc9pgc9s8qqaq=dirpe0xb9q8qiLsFr0=vr0=vr0dc8meaabaqaciaacaGaaeqabaqabeGadaaakeaacqWGzbqwdaWgaaWcbaGaemitaWeabeaakiabg2da9maalaaabaGaemywaK1aaSbaaSqaaiabigdaXaqabaGccqGHsislcqWGlbWsdaWgaaWcbaacciGae8xSdegabeaakiabcIcaOiabdggaHnaaBaaaleaacqaIXaqmaeqaaOGaey4kaSIaemyyae2aaSbaaSqaaiabikdaYaqabaGccqWGzbqwdaWgaaWcbaGaeGymaedabeaakiabc+caViabikdaYiabcMcaPaqaaiabigdaXiabgUcaRiabdUealnaaBaaaleaacqWFXoqyaeqaaOGaemyyae2aaSbaaSqaaiabikdaYaqabaGccqGGVaWlcqaIYaGmaaGaeiilaWIaaCzcaiaaxMaadaqadiqaaiabiodaZaGaayjkaiaawMcaaaaa@4FFD@,

where *K*_*α *_is a constant corresponding to the probability interval α (see Additional file 1).

Three reliability checks were incorporated into the consecutive sampling program. First is the identity test, which verifies the equality

*SD*(*Y*_*diff*_) = *SD*(*Y*_1_) +*SD*(*Y*_2_),     (4)

where *SD*(*Y*_*diff*_) and *SD*(*Y*_*i*_) are the standard deviations calculated from the expression difference and from the expression values of the individual (first or second) arrays, respectively [10]. It provides a good verification of variability of the mean values within samples; we usually require the mean discrepancy of the ten consecutive samples below 1%. The second reliability check calculates the average number of samples failing the Kolmogorov-Smirnov normality test (P = 0.05) and the third compares the number of genes beyond the 0.95 probability interval to the number, corresponding to the same interval of the normal distribution with the same mean and standard deviation.

## Competing interests

The author(s) declare that they have no competing interests.

## Authors' contributions

JPN conceived the study, developed the methods and computer programs, performed most of the evaluations and prepared the first draft of the manuscript. CW and JPN collaborated on preparation of the final version of the article and S-YK participated extensively on evaluation of the data. S-YK, OM, DH and JS significantly contributed during the revisions of the text and provided the data; several datasets were provided by CW. JX also provided help with the final formatting. Remaining authors provided the data and other relevant information and participated on the revisions.

## Reviewers' comments

### Reviewer's report 1

*Yoav Gilad, Dept. of Human Genetics, University of Chicago, 920 E. 58th Street – CLSC 325C, Chicago, IL 60637, USA (nominated by Doron Lancet, Department of Molecular Genetics, Weizmann Institute of Science, Rehovot 76100, Israel)*.

I agree with the authors that it is important to characterize the dispersion – along with other properties of microarray data. I also agree that many of us in this field are analyzing our data without being properly aware of the assumptions we make. In that respect, the presented analysis is useful and the results are reassuring. If I am not mistaken, Gary Churchill has previously demonstrated that normality is a valid assumption for expression data, and his work should be cited here.

I am not a statistician and hence do not feel qualified to comment on the details of the statistical analysis presented in this manuscript (I recommend that it will be seen by at least one statistician prior to publication). However, I do question the validity and relevance of the analysis of the PM-MM signals. While the rationale presented in the paper (not different than what is claimed by Affymetrix) is clear, empirical observations (including in my own group) suggest that in many cases nearly all the binding – both to the PM as well as to the MM – is of the specific RNA of interest. In those cases, the power to 'detect' expression, as well as the power to estimate non-specific hybridization, is weak. Moreover, in many cases (again-including in our hands), negative PM-MM values were observed while the expression of specific RNA of interest could be demonstrated by other means (such as RT-PCR). I believe that work by others (mostly cited by the authors) demonstrated that the power to detect differential expression is higher when PM-only estimates are considered. Perhaps studying the properties of PM-only data will be proven more useful.

**Author Response**: *First we would like to thank Dr. Gilad for his review and for bringing up an interesting issue of the MM signals. Regarding the question of normal distribution, we looked over Dr. Churchill's papers dealing with microarray technology, including Cui et al. Biostatistics (2005) [a], **6**:59–75, Cui and Churchill, Genome Biology (2003), **4**:210 [b], Churchill, BioTechniques (2004), **37**:173 [c], Kerr, Churchill. Genet Res. (2001), **77**:123 [d], Kerr, Churchill (2001), PNAS, **98**:8961 [e], Kerr et al. (200), J. Comp. Biol., **7**:519 [f], but we did not find confirmation of normality in dispersion of single-color microarrays. In case of the spotted two-color microarrays the authors detected non-normal distribution of residues [d-f]*.

*Regarding the question of usefulness of MM signals: Our main objective is to show that the dispersion across all types of arrays, experimental conditions and organisms exhibits some common basic properties and we did not intend to make a case in favor of the Affymetrix approach. Nonetheless, we feel that the fact that such common description can be found supports reasoning of the Affymetrix*.

*The discussion is still on and various arguments have been brought up both for and against using the MM signals. We fully agree with the reviewer that a major part of the signal of MM probes is due to the "specific" RNA of interest. However, this is to be expected, since among all RNA molecules attaching to the MM probe, the particular RNA of interest is most likely closest to its structure. It is exactly the ability to distinguish between the perfect match and "almost-perfect-match" that makes the measured signal reliable. If a substantial quantity of the specific RNA is present and the signals of both PM and MM are equal or MM exceeds PM, it suggests either saturation or low distinguishing power. Under such circumstance the MM signal provides useful information, indicating that the particular PM signal might not reflect the true RNA concentration. In case of saturation, taking the PM signal only would correctly indicate that the specific RNA is present, however, the relationship abundance-signal would be strongly nonlinear. As we mention in the Discussion, the results of various studies aiming at validation of different approaches are inconclusive. Evidently, more research is needed to establish the optimal technology and corresponding statistical procedures. It is likely that no single methodology could be found universally optimal and different circumstances would call for different approaches*.

### Reviewer's report 2

Sach Mukherjee, Department of Statistics, University of California, Berkeley, CA, 94720-3860, USA (nominated by Sandrine Dudoit, Division of Biostatistics, School of Public Health, University of California, Berkeley, CA 94720-7360, USA)

The authors present an empirical study of the distributional characteristics of data from Affymetrix gene expression microarrays. One of the questions posed at the outset concerns the relationship between the mean and variance of microarray data ("it is useful to know how the standard deviation behaves across the expression range...", [Background, §2]) and is subsequently answered in the following way: "...the standard deviation is linearly proportional to the mean expression level" [Results, Frequency distributions, §3]. However, this latter finding seems widely recognized already, and has been discussed in some detail in the literature (e.g. Rocke and Durbin, 2001; Durbin et al. 2002; Huber et al. 2002). Yet none of these papers are cited in the article.

**Author Response**: *We agree that the fact that the standard deviation in the high region is proportional to the signal and at the low end it does not converge to zero has been generally accepted, but we are not aware of the study that systematically verified the linear relationship. Rocke and coworkers derived a similar model from theoretical considerations and corresponding references were included*.

The authors also criticize the use of log-transformation [Background, §1] (again without referring to the literature on the topic) but then seem to use just such a transformation as a pre-processing step before regression [Results, Consecutive sampling analysis, §1]. Yet under a data model with both multiplicative and additive noise, data are only log-normally distributed at high expression levels (Durbin et al. 2002). Furthermore, log-transformation may inflate the variance of observations with low expression levels. Indeed, the authors find that "...larger percentages of failures [in passing a K-S test of Normality] occur in the near-zero region." [Results, Frequency distributions, §1]. Might not this effect simply be due to the log-transformation?

**Author Response**: *It appears that our procedure was not clearly described and we revised the text accordingly. We actually use the logarithmic transform only in the regression procedure to balance the residuals, i.e. to prevent the residuals of the high-expression genes to outweigh the low-expression range. Thus instead of regressing*

*SD*(*Y*_*mean*_) → *a*_1 _+ *a*_2_*Y*_*mean*_

we fit

log(*SD*(*Y*_*mean*_)) → log(*a*_1 _+ *a*_2_*Y*_*mean*_)

*Consequently, the determined characteristic function represents the standard deviation of the original (normalized) data and not log-transformed data. This is the only occasion when we use the log-transform, in all other procedures we employ non-transformed normalized data*.

This reviewer found the approach taken to "consecutive sampling" in studying the mean-variance relationship in paired arrays somewhat ad hoc. For example, the 250 probe sets having highest expression level are excluded from the analysis. What effect does this exclusion have on the analysis? Is it appropriate to leave out data (arguably some of the most interesting data) from an empirical study of this kind? This issue is not really discussed. The authors also state that "we usually require the mean discrepancy of the ten consecutive samples below 1%" [Methods, §1]. Does this mean the data are ignored if the discrepancy is higher than this threshold?

**Author Response**: *The consecutive samples provide reliable representation of the standard deviation only if the within sample differences of means are small, say below 1%. At the maximum of the expression range the density of the points is small and, consequently, differences in the mean values are large. To obtain dependable coefficients of the standard deviation function these data have to be excluded from the regression procedure. Subsequently, we assume that validity of the standard deviation characteristic function can be extrapolated to the maximum expression value. Indeed, in the differential expression analysis all data to the maximum expression are included. The text was revised to avoid misunderstanding*.

*Regarding a discrepancy in identity (4): The consecutive sampling program automatically keeps track on assumptions and signals detected problems. Identity (4) is a convenient check of the variability of means and, generally, of the reliability of characteristic function; it is typically fulfilled within 0.1%. If the difference between the right-hand and left-hand sides of the equation exceeds 1% the program raises a flag, indicating problematic data. In such case the researcher conducting analysis examines the data and determines the reason for discrepancy; if no corrective measure can be found, the sample is excluded from the analysis*.

The presentation of mathematical details is not always very clear in the paper. Equations (2) and (3) would benefit from either a derivation or a reference. Equally, some of the phrasing is somewhat difficult to interpret, e.g. "...we can calculate an estimator of the standard deviation of gene expressions variability of a population of replicate arrays from two-array comparisons" [Results, Consecutive sampling analysis, §1 (the text before revision)].

**Author Response**: *Derivation of equations (2) and (3) is described in *Additional file 1. *The sentence in question was reformulated*.

Finally, this reviewer found the introduction of a new methodology for finding differentially expressed genes [Results, Probability intervals and correlation of the *K*_*α *_coefficients with *t-*distribution, §5 onwards] puzzling inasmuch as it did not relate to, or strengthen, any of the main arguments of the paper. The case presented was also far from convincing: given that there are so many existing methods for detecting differential expression, it is surely reasonable to expect any new method to be accompanied by strong empirical evidence and/or theoretical arguments in its favor.

**Author Response**: *Actually, the application of the probability intervals to the differential expression analysis had not been included in the earlier versions of the manuscript. However, during the internal reviews we frequently encountered a question "how can the dispersion analysis and probability intervals help biologist to analyze data and to detect significant differences in gene expression" (see also comment of the third referee). To answer this question we included a brief description of the consecutive sampling and coincidence analysis, which we use as a standard procedure, usually in combination with the RMA and/or other approaches. To provide better description we revised the text and included an additional reference*.

In conclusion, the basic idea behind the paper, of characterizing microarray data distributions using a large set of real-life experimental data, is a very good one, but the paper is not well tied to the literature and suffers at times from a somewhat ad hoc approach.

References:

Rocke DM, Durbin B. Approximate variance-stabilizing transformations for gene-expression microarray data. Bioinformatics. 2003 May 22; 19 (8):966-72.

Durbin BP, Hardin JS, Hawkins DM, Rocke DM. A variance-stabilizing transformation for gene-expression microarray data. Bioinformatics. 2002; 18 Suppl. 1:S105-10.

Huber W, von Heydebreck A, Sultmann H, Poustka A, Vingron M. Variance stabilization applied to microarray data calibration and to the quantification of differential expression. Bioinformatics. 2002; 18 Suppl. 1:S96-104.

### Reviewer's report 3

Amir Niknejad and Shmuel Friedland, Department of Mathematics, Statistics and Computer Science University of Illinois at Chicago 851 S. Morgan Street Chicago, IL 60614 USA (nominated by Neil Smalheiser, Department of Mathematics, Statistics and Computer Science, University of Illinois at Chicago, 851 S. Morgan Street, Chicago, IL 60614, USA)

The paper addresses issues related to analysing DNA Microarrays data focusing on differences of gene expression. The paper is an extension of previous paper of J.P. Novak (reference# 8) by employing various parametric and nonparametric statistics tools and extensive use of statistical packages for very large data sets. The premise of the paper is that the standard deviation of samples of difference of gene expression in DNA microarrays is a linear function of their mean. The paper is a very good work in the area of quality control of Data in DNA Microarrays and certainly a contribution to the field. There are several points that the authors should clarify:

1. The authors mentioned that "majority of microarray samples (85%–95%) conform to a Gaussian distribution". What is the reason for the rest of 5%–15% of microarrays sample which do not conform with normality? Is it a biological reason or just manufacturing technology problem?

**Author response**: *We thank Dr. Niknejad and Dr. Friedland for the very helpful review. In response to the question above: According to our extensive experience with the Affymetrix arrays and limited experience with the Illumina fiberoptic bead-based oligonucleotide microarrays, the manufacturing technology is an unlikely reason. The outliers, the most frequent cause of non-normal distribution, are probably caused by random fluctuations in the experimental procedures, such as hybridization or labeling. A discontinuity in the frequency distribution (i.e. one part of the curve having systematically higher coefficient of amplification than the other) or its derivative is difficult to explain. (Note that the number of the cited reference by Novak et al. was changed from 8 to 10.)*

2. The authors mentioned that "filtering 15% of genes would enhance the performance for clustering methods". The question is how is this filtering being done, and what is its effect on the data set as a whole and the biological ramification of it.

**Author response**: *Generally, all clustering methods are sensitive to noise, however, the problem is more difficult in unsupervised clustering, where members of presumed classes are unknown. Hierarchical clustering and principal component analysis appear to be among the most sensitive, while self organized maps are more robust. Approach to the problem and optimal percentage of the probe sets filtered out depends on a given set of data. Actually, we did not specify percentage in the text – fifteen percent is relatively low and should refer to the set used for analysis, usually reduced by eliminating probes with overall near-zero values. For the clustering procedures we raise the required threshold and try to identify "informative" probe sets, i.e. probe sets likely to be characteristic for presumed classes. Very small groups are virtually impossible to discover in noisy data, so we assume some minimum number k of samples in any particular group – say k ~ 5. Then we select only the probe sets, with k or more expression at least r-fold larger or s-fold smaller than the total median; typically 2 < r, s < 5. It is important to repeat the clustering procedure for several sets of parameters to ensure that the identified classes are independent of filtering constanst*.

We are also concerned with mostly focusing on Affymetrix technology for coming up for means of quality control receipt. It will be a good idea to see how their model fares for other brands of microarrays.

**Author response**: *Our experience is limited to the Affymetrix and Illumina microarrays. However, in our opinion, it is likely that dispersion characteristics of all single-color arrays are similar*.

It would be helpful if the authors mention how their findings can help molecular biologists to make inferences about gene expression data of various microarray data sets and their biological implications.

**Author response**: *Beside filtering of the randomly variable probe sets in noisy data the most practical application is combination of the consecutive sampling analysis and coincidence testing applied to evaluation of the observed differences between experiment and control arrays. The unique advantage of this approach is that it can be applied to assays with small number of replicates (two or more). It is a nonparametric method, theoretically equivalent to repeated random selection, and it is easy to estimate the probability of false positives. Moreover, it is based on pair-wise comparisons and it enables automatic detection of problematic arrays. We extended the discussion of this application in the section Results and added two references*.

## Supplementary Material

Additional File 1 Derivation of equalities (2) and (3). Click here for file
