# Supplementary material for: Generalization of DNA microarray dispersion properties: microarray equivalent of t-distribution
Source: Biol Direct. 2006 Sep 7;1:27. doi: 10.1186/1745-6150-1-27 (PMC1586001; doi:10.1186/1745-6150-1-27)
Supplement: Additional File 1 — Derivation of equalities (2) and (3). [file 1745-6150-1-27-S1.doc]

### Additional file 1 – Equalities (2) and (3)

File format: Microsoft Word

Derivation of equalities (2) and (3).

When defining the boundaries of a given probability interval we require that the differences of all expression values are within a constant number of standard deviations, i.e. for any values *Y_1_* and *Y_2_* we have a condition

$-K_{\alpha}SD<Y_{2}-Y_{1}<K_{\alpha}SD$. (A1)

Using the condition (A1) we can express the upper limit of *Y_2_* in terms of *Y_1_* as

$Y_{U}=K_{\alpha}SD+Y_{1}$. (A2)

Substituting for SD the expression

$SD=a_{1}+a_{2}\frac{Y_{1}+Y_{2}}{2}$ (A3)

we obtain

(A4)

and subsequently

$Y_{U}=\frac{Y_{1}+K_{\alpha}(a_{1}+a_{2}Y_{1}/2)}{1-K_{\alpha}a_{2}/2}$. (A5)

Similarly for the lower limit we write

$Y_{L}=K_{\alpha}SD+Y_{1}$ (A6)

and, finally,

$Y_{L}=\frac{Y_{1}-K_{\alpha}(a_{1}+a_{2}Y_{1}/2)}{1+K_{\alpha}a_{2}/2}$. (A7)
